# Phospho-selective mechanisms of arrestin conformations and functions revealed by unnatural amino acid incorporation and ^19^F-NMR

**DOI:** 10.1038/ncomms9202

**Published:** 2015-09-08

**Authors:** Fan Yang, Xiao Yu, Chuan Liu, Chang-Xiu Qu, Zheng Gong, Hong-Da Liu, Fa-Hui Li, Hong-Mei Wang, Dong-Fang He, Fan Yi, Chen Song, Chang-Lin Tian, Kun-Hong Xiao, Jiang-Yun Wang, Jin-Peng Sun

**Affiliations:** 1 Laboratory of Quantum Biophysics and Laboratory of RNA Biology, Institute of Biophysics, Chinese Academy of Sciences, 15 Datun Road, Chaoyang District, 100101, China; 2 Key Laboratory Experimental Teratology of the Ministry of Education and Department of Biochemistry and Molecular Biology, Shandong University School of Medicine, 44 Wenhua Xi Road, Jinan, Shandong 250012, China; 3 Department of Physiology, Shandong University School of Medicine, Jinan, Shandong 250012, China; 4 Department of Pharmacology, Shandong University School of Medicine, Jinan, Shandong 250012, China; 5 Department of Biochemistry, University of Oxford, Oxford OX13QU, UK; 6 Hefei National Laboratory for Physical Science at Microscale and School of Life Science, University of Science and Technology of China, Hefei, Anhui 230027, China; 7 Department of Pharmacology and Chemical Biology, School of Medicine, University of Pittsburgh, Pittsburgh, Pennsylvania 15261, USA; 8 Department of Medicine, School of Medicine, Duke University, Durham, North Carolina 27705, USA

## Abstract

Specific arrestin conformations are coupled to distinct downstream effectors, which underlie the functions of many G-protein-coupled receptors (GPCRs). Here, using unnatural amino acid incorporation and fluorine-19 nuclear magnetic resonance (^19^F-NMR) spectroscopy, we demonstrate that distinct receptor phospho-barcodes are translated to specific β-arrestin-1 conformations and direct selective signalling. With its phosphate-binding concave surface, β-arrestin-1 ‘reads' the message in the receptor phospho-C-tails and distinct phospho-interaction patterns are revealed by ^19^F-NMR. Whereas all functional phosphopeptides interact with a common phosphate binding site and induce the movements of finger and middle loops, different phospho-interaction patterns induce distinct structural states of β-arrestin-1 that are coupled to distinct arrestin functions. Only clathrin recognizes and stabilizes GRK2-specific β-arrestin-1 conformations. The identified receptor-phospho-selective mechanism for arrestin conformation and the spacing of the multiple phosphate-binding sites in the arrestin enable arrestin to recognize plethora phosphorylation states of numerous GPCRs, contributing to the functional diversity of receptors.

## Abstract (web summary)

G-protein-coupled receptors (GPCRs) signal via G proteins or arrestin-mediated pathways; the plasticity of arrestin proteins is thought to underlie their function. Here, the authors use NMR to examine how β-arrestin-1 recognizes different GPCR phospho-barcodes, and how this triggers structural rearrangements to fulfill selective functions.

## Graphical abstract

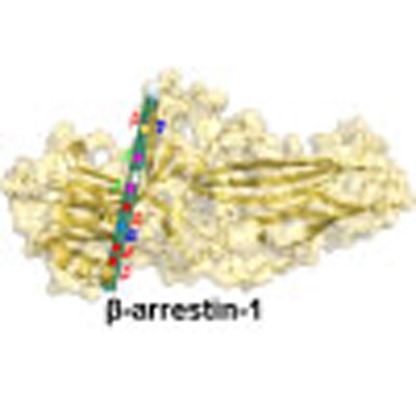

G-protein-coupled receptors (GPCRs) convert extracellular stimuli to intracellular signalling cascades primarily through G proteins or arrestin-mediated pathways^1^,^2^,^3^,^4^. G proteins transduce signals by regulating the levels of second messengers, whereas arrestins recruit distinct downstream proteins to either desensitize receptors or initiate their own signalling pathways^5^,^6^,^7^,^8^,^9^,^10^. Recently, significant conformational changes in arrestin have been observed following specific phosphopeptide binding or the formation of a receptor/arrestin complex. For example, the crystal structure of the V2-vasopressin receptor carboxy-terminal–phosphopeptide (V2R–phosphopeptide (V2Rpp))/β-arrestin-1 complex revealed that the binding of V2Rpp induced the rotation of the amino domain of β-arrestin-1 with respect to its C-terminal domain^11^. In another study, data obtained by electron microscopy (EM) and hydrogen–deuterium exchange mass spectrometry studies revealed increased dynamics in both the N- and C-terminal domains of β-arrestin-1 after β2-adrenergic receptor (β2AR)/β-arrestin-1 complex formation^2^. These results suggest that the structural plasticity of β-arrestins underlies their important cellular functions.

Arrestins are multi-functional proteins^7^,^12^. Previous studies have indicated that two distinct features of these proteins—ligand-induced receptor conformation and receptor phosphorylation barcodes—contribute to the specific arrestin conformations that dictate selected arrestin functions^5^,^13^. Questions regarding these elements are core issues in the study of signal transduction by GPCRs, in particular given the plethora of phosphorylation states and receptor conformations of numerous receptors^5^,^14^,^15^,^16^,^17^. However, the precise mechanism by which arrestin conformation is determined based on either a ligand-induced receptor-specific conformation or a selective phospho-barcode remains uncertain. Moreover, various receptors have no defined phosphorylation sequence information that correlates with their distinct arrestin-mediated functions, despite the existence of a myriad of evidence supporting the essential roles of phosphorylation and of negatively charged residues in the cytoplasmic regions of receptors in arrestin-mediated receptor endocytosis and other functions^14^,^16^,^17^,^18^. These findings raise the question of whether specific phospho-barcodes exist to direct barcode-selective arrestin functions. If such barcodes exist, then the method by which they are decoded by arrestins and translated into particular arrestin conformations remains unknown. The structural heterogeneity and flexibility of active arrestins have hampered the characterization of arrestin conformations by crystallography or EM, and all active arrestin conformations determined to date have been obtained by stabilizing arrestin complexes with conformationally selective antibodies^2^,^11^. Therefore, it is desirable to develop alternative approaches to detect conformational changes in arrestins and decipher the phospho-selective mechanisms underlying distinct arrestin functions.

Recently, site-directed fluorine-19 nuclear magnetic resonance (^19^F-NMR) spectroscopy has been used as a powerful approach for characterizing the dynamic conformational changes of large signalling protein complexes or membrane proteins^1^. In addition, we have developed an efficient method for incorporating the unnatural amino acid 3,5-difluorotyrosine (F2Y) into proteins by expanding the genetic code of *Escherichia coli*^19^,^20^. In the present study, by using this method to incorporate F2Y at specific locations in β-arrestin-1, we demonstrated that β-arrestin-1 reads receptor phospho-C-tail messages with its phosphate-binding concave surface, which harbours at least ten potential phosphate-binding sites (numbered 1–7 according to the binding mode of V2Rpp to β-arrestin-1 in the complex structure of V2Rpp/β-arrestin-1, with A1–A3 indicating additional phosphate-binding sites). Although all functional phosphopeptides interact with phosphate-binding site 1 of β-arrestin-1, different GRK–phosphopeptides (GRKpps) interact with β-arrestin-1 through distinct phospho-interaction patterns that are coupled to selective cellular functions. Whereas GRK2–phosphopeptides (GRK2pps) interact with β-arrestin-1 within a phospho-binding sequence of the 1-4-6-7 pattern and promote clathrin recruitment, the GRK6–phosphopeptide (GRK6pp) interacts with β-arrestin-1 through a different 1–5 sequence and provides the SRC signalling order. Moreover, ^19^F-NMR spectra provide direct biophysical evidence for the presence of at least three unique active β-arrestin-1 structural states corresponding to different phospho-binding patterns and only one of these structural states is recognized by clathrin. In cells, the mutation of key residues in specific phosphate-binding sites selectively abolished different β-arrestin-1 functions downstream of several receptors, suggesting that the identified phospho-pattern-selective mechanism of β-arrestin-1 conformation and function might be common to at least a subset of GPCR family members. Our results provide mechanistic insight into how the structural plasticity of β-arrestin-1 enables its recognition of specific phospho-patterns and translates these phospho-messages into distinct structural rearrangements and functional outcomes.

## Results

### The development of ^19^F-NMR probes of β-arrestin-1

**Figure 1:**
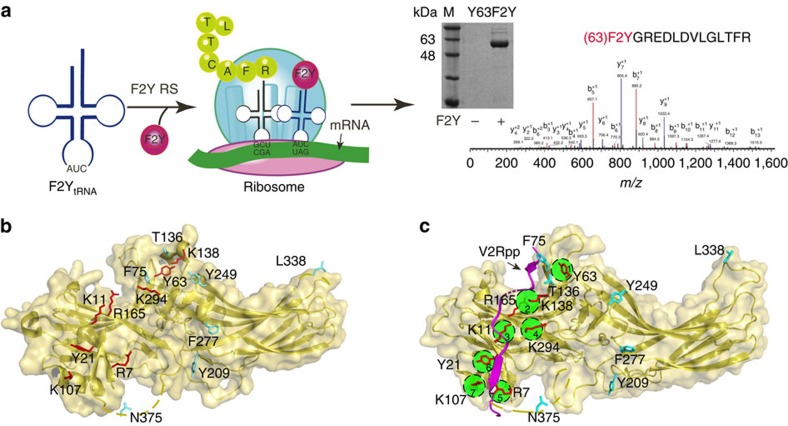
The development of ^19^F-NMR probes via the incorporation of F2Y at specific positions of β-arrestin-1. (**a**) Schematic flowchart of the incorporation of F2Y into β-arrestin-1. For example, the β-arrestin-1–Y63-F2Y protein was acquired by co-transfection of β-arrestin-1 mutant plasmid and the pEVOL-F2YRS plasmid encoding specific *Methanocaldococcus jannaschii* tyrosyl amber suppressor transfer RNA/tyrosyl-tRNA synthase mutants with F2Y in the culture medium. The purity of the protein was determined by electrophoresis (middle panel). The purified protein was subjected to trypsin digestion and analysed by MS/MS spectroscopy, which indicates the presence of the F2Y-G-R fragment, MW 413, b_3_^+1^, F2Y-G-R-E-D, MW 657 and b_5_^+1^, for example. These results confirmed that F2Y was incorporated into β-arrestin-1 at Y63. *m*/*z*, mass/charge ratio. (**b**) Frontal view of the F2Y incorporation sites in the inactive β-arrestin-1 crystal structure (PDB:1G4M, yellow). F2Y incorporation sites as phospho-interaction probes (R7, K11, Y21, Y63, K107, K138, R165 and K294) that directly interact with V2Rpp are shown in red. F75 in the finger loop, T136 in the middle loop, F277 in the lariat loop, Y209 between β strands XIII and XIV, Y249 between β strands XV and XVI, L338 in the splice loop and N375 adjacent to the classic clathrin-binding box are shown in cyan. The dashed lines depict the structural portion not resolved by crystallography. (**c**) F2Y incorporation sites in the active β-arrestin-1 structure (PDB: 4JQI). The bound V2Rpp is shown in magenta. The seven phosphate-binding sites are indicated by green circles.

**Figure 2:**
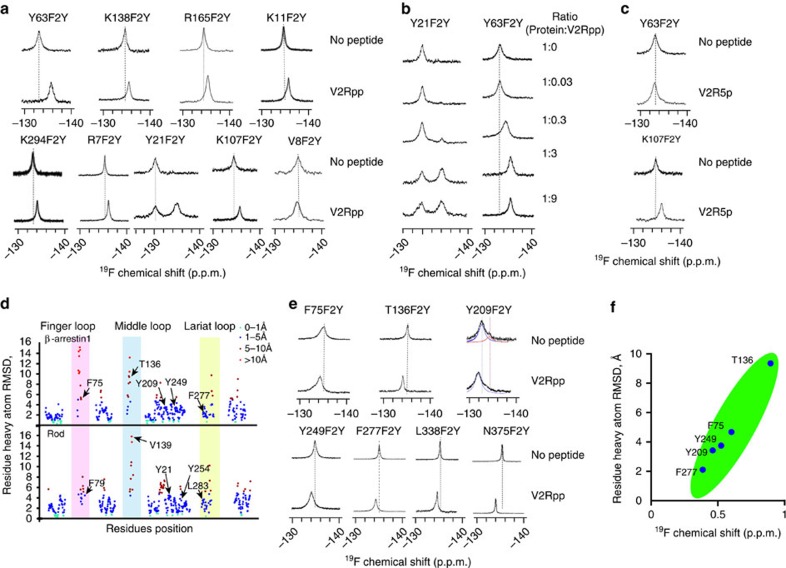
Application of ^19^F-NMR probes of β-arrestin-1 allowed the detection of phosphate binding and structural changes by ^19^F-NMR spectroscopy. (**a**) Upfield shifts in the ^19^F-NMR spectra of F2Y-phospho-sensing-probes of β-arrestin-1 are observed in response to V2Rpp binding. The chemical shifts are referenced to trifluoroacetic acid (TFA, −76.5 ppm) as an internal standard. (**b**) ^19^F-NMR spectra of β-arrestin-1–Y21-F2Y and β-arrestin-1–Y63-F2Y after titration with V2Rpp. Left: two distinct peaks were observed for Y21 after titration with V2Rpp. The peak at −135.216 p.p.m. increased (representing the V2Rpp-bound state) as the peak at −130.181 p.p.m. (representing the state of β-arrestin-1 alone) decreased, indicating a slow conformational exchange. Right: a single peak was observed for Y63; the chemical shift varied in response to differences in the concentration of the V2Rpp, suggesting that rapid conformational changes occur at this site after V2Rpp binding. (**c**) Binding of V2R5p, which lacks the first three phosphates in V2Rpp (pT347, pS350 and pS357), caused an upfield shift at the K107-F2Y position but no detectable change at the Y63-F2Y position (Δp.p.m.<0.05). (**d**) Plots of the distance root mean square deviations (RMSDs) for individual residues between inactive β-arrestin-1 and the V2Rpp-bound β-arrestin-1 complex (upper panel) and between inactive rod arrestin and its constitutively active p44 variant (lower panel). The vertical axis shows all heavy-atom RMSDs per arrestin residue. The colour code shown in the upper right corner of the panel indicates the Cα deviation of each residue. The three loops that exhibit major conformational changes are highlighted: finger loop, red; middle loop, light blue; and lariat loop, light yellow. (**e**) The ^19^F-NMR spectra of β-arrestin-1 incorporating site-directed F2Y revealed dynamic conformational changes after activation by V2Rpp. (**f**). Heavy-atom RMSD versus the relative amplitude of the ^19^F-NMR chemical shift for specific F2Y incorporation sites.

The seven phosphate-binding sites revealed from the crystal structure of the V2Rpp/β-arrestin-1 complex allowed the identification of at least 127 potential phosphate-interacting patterns. It is useful to develop phosphate-binding sensors to reveal how β-arrestins fine-tune the phospho-pattern from a large number of receptors^11^. Accordingly, we selected eight positions that covered all phosphate-binding sites in the crystal structure of the V2Rpp/β-arrestin-1 complex at which to incorporate F2Y (Fig. 1a–c and Supplementary Figs 1–4). The ^19^F-NMR spectra revealed two types of conformational states after V2Rpp binding: a slow exchange state (Y21) and a fast exchange state (Y63 and all other F2Y-incorporated positions) (Fig. 2a,b and Supplementary Tables 1–4). Paramagnetic titration experiments suggested that V2Rpp binding induced a structural state with greater solvent accessibility at the Y21 position (Supplementary Fig. 5). The phosphopeptide concentration dependence of the chemical shift of F2Y-Y63 and the increased area of the upfield shift peak of Y21-F2Y are consistent with concentration-dependent clathrin recruitment in the glutathione *S*-transferase (GST) binding assay, indicating the existence of a good structure–function relationship (Supplementary Fig. 6 and Supplementary Table 3). In particular, all F2Y positions that directly interacted with phosphate displayed an upfield shift after V2Rpp binding (Fig. 2a and Supplementary Tables 1 and 2). By contrast, the binding of V2Rpp to V8-F2Y, which localizes to the β-strand I of arrestin and forms hydrophobic interactions with L365 of the V2Rpp, displayed a downfield shift in the ^19^F-NMR spectra (Fig. 2a). In addition, the binding of V2R5p to β-arrestin-1 caused an upfield shift of the ^19^F-NMR spectra at K107-F2Y but not at Y63-F2Y (Fig. 2c and Supplementary Table 2). Therefore, these F2Y probes may serve as phospho-pattern detectors to monitor the increasing negative charges after the binding of phosphorylated receptor or nucleic acid molecules^21^,^22^.

To study phosphopeptide-induced arrestin conformational changes, we next chose residues for F2Y incorporation that do not directly interact with V2Rpp (Fig. 1b,c and Supplementary Figs 7 and 8). Active crystal structures of the V2Rpp/β-arrestin-1 complex and a rod-arrestin splicing variant, p44, were compared with their inactive structural partners, and the results revealed a common pattern of differences^11^,^23^ (Fig. 2d). In addition to selecting residues that might exhibit broad conformational changes after activation, we attempted to study positions that are naturally occupied by Y or F to minimize the effects of mutation. All of the substitutions were tested using cellular and *in vitro* analyses to ensure that arrestin functions were not affected (Supplementary Figs 4 and 9–12). The ^19^F-NMR spectra of β-arrestin-1 alone at the Y209-F2Y position revealed a state of slow exchange between two peaks; these peaks were reduced to a single peak after V2Rpp binding (Fig. 2e). Furthermore, the amplitude of the ^19^F-NMR chemical shifts induced by V2Rpp binding at the F2Y-incorporated sites increased in the order F277, Y209, Y249, F75 and T136, and these increases were proportional to the increased root mean square deviation differences between the crystal structures of the β-arrestin-1/V2Rpp complex and β-arrestin-1 alone at the corresponding positions (Fig. 2e,f and Supplementary Tables 1 and 3). Although the chemical shift and root mean square deviation are different variables, both increased together, reflecting a substantial structural rearrangement at these positions following arrestin activation. Therefore, the insertion of F2Y at these positions can be used as a ^19^F-NMR probe to detect dynamic arrestin conformational changes.

### GRK phosphorylation determines β-arrestin-1 function

**Figure 3:**
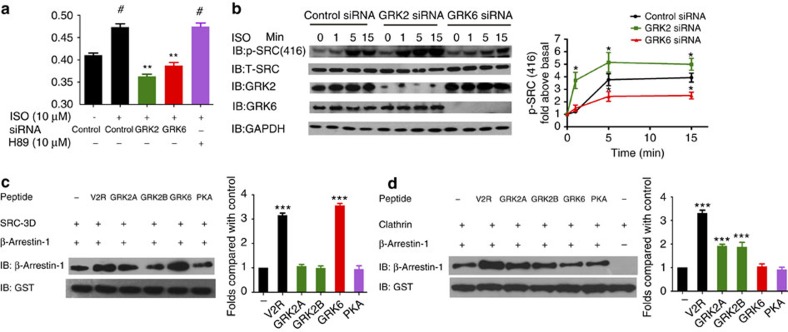
Different phospho-barcodes encode different arrestin functions and conformations. (**a**) Activation of β2-AR-induced conformational changes in β-arrestin-1 as measured using intramolecular BRET. Plasmids encoding a β-arrestin-1 BRET biosensor (Luc-β-arrestin-1-YFP) and Flag-β2-AR were transiently co-transfected in HEK293 cells. After starvation, the cells were stimulated with ISO (10 μM) for 10 min. The effects of GRK phosphorylation of β2-AR on ISO-induced β-arrestin-1 conformational change were probed using siRNA-mediated GRK knockdown. (**b**) A representative western blotting is shown for the effects of knockdown of specific GRKs in β2-AR-mediated proto-oncogene tyrosine-protein kinase (SRC) activation. Left panel: HEK293 cells that were transiently transfected with Flag-β2-AR were stimulated with ISO for 1, 5 and 15 min. To examine the roles of GRK2 and GRK6 in β2-AR-mediated SRC activation, siRNAs that were selective for specific GRKs were transfected into the cells. A western blot representative of at least three independent experiments is shown on the left. Right panel: western blot signals of phospho-SRC 416 (**a**) were quantified using densitometry and are expressed as fold change versus basal level. Whereas GRK6 is required for SRC activation downstream of β2-AR activation, GRK2 is a negative regulator of β2-AR-induced SRC activation. (**a**,**b**) At least three independent experiments were performed. #*P*<0.05, ISO-stimulated cells are compared with unstimulated cells. *P<0.05; **P<0.01; ***P<0.005; GRK siRNA-treated cells are compared with control siRNA-treated cells. (**c**,**d**) Effects of phospho-peptide binding on β-arrestin-1/clathrin complex formation (**c**) or β-arrestin-1/SRC-SH2-SH3-kinase-three domain (SRC3D) complex formation (**d**). β-arrestin-1 (300 nM) was incubated with an equal concentration of various phosphopeptides and either GST-clathrin 1–494 (**c**) or GST-SRC3D (**d**). The complexes were pulled down using GST beads and the amount of β-arrestin-1 bound to clathrin or SRC3D was monitored using a specific β-arrestin-1 antibody (left panel in each figure). At least three independent experiments for each assay were performed. Western blot signals of β-arrestin-1 bound to clathrin (**c**) or SRC3D (**d**) from the left panels were quantified using densitometry and are shown as columns (right panel). **P<0.01; ***P<0.005; phosphopeptide stimulated β-arrestin-1/clathrin complex formation (**c**) or β-arrestin-1/SRC complex formation (**d**) are compared with the basal state.

The individual receptor seven-transmembrane core and specific phosphorylation patterns are two determinants of specific arrestin functions^2^,^5^,^13^. The GRK6-phosphorylated β2AR imparts β-arrestin-2 conformations that specifically activate ERK2, whereas GRK2-phosphorylated β2AR exerts more pronounced effects on receptor internalization (Table 1)^5^,^16^,^24^,^25^. Although these studies demonstrated that the functions of β-arrestin-2 are defined by different GRKs in the cells, whether the conformation and signalling of β-arrestin-1 are regulated by selective GRK-encoded receptor phosphorylation pattern has not been examined. Therefore, we generated a BRET sensor (Rluc-β-arrestin-1-YFP) to monitor the conformational change of β-arrestin-1 in cells. Stimulation with 10 μM isoproterenol (ISO) significantly increased the BRET signal of Rluc-β-arrestin-1-YFP, indicating a conformational change of β-arrestin-1 after β2AR activation (Fig. 3a). However, the ISO-induced BRET signal was significantly reduced by the knockdown of either GRK2 or GRK6, indicating important roles of GRK in mediating the β-arrestin-1 conformational change after receptor activation (Fig. 3a).

We next investigated whether receptor phosphorylation by different GRKs produced different signalling outcomes downstream of β-arrestin-1. Formation of the receptor/β-arrestin-1/SRC (or SRC family member HCK, FGR or YES) ternary complexes has been demonstrated to regulate multiple cellular functions downstream of various receptors, including, but not limited to, anti-apoptotic effects, granule exocytosis, colorectal carcinoma cell migration, bacterial adhesion to endothelial cells and the proliferation of pancreatic β-cells^26^,^27^,^28^,^29^,^30^,^31^. Therefore, we inspected the roles of different GRKs in β-arrestin-1-mediated SRC activation. Whereas GRK6 knockdown significantly decreased SRC activation downstream of β2AR activation, GRK2 knockdown increased SRC activation (Fig. 3b). Together with previous research, these experiments demonstrate that GRK2 and GRK6 encode distinct β-arrestin-1 conformations and functions in cells^5^,^8^,^28^,^29^,^30^,^32^.

To further evaluate whether these distinct cellular outcomes regulated by different GRKs are due to the generation of specific phosphorylation information in the receptor C-tail that is decoded by β-arrestin-1, we synthesized specific GRK2- or GRK6-phosphorylated β2AR C-tails and examined their effects on the biochemical properties of β-arrestin-1 (Table 1). The GRK2-phosphorylated β2AR C-tail were separated into two segments, GRK2App and GRK2Bpp, owing to difficulties encountered with synthesis. Protein kinase A (PKA) also phosphorylates β2AR and we used the PKA-phosphorylated β2AR peptide (PKApp) as a control, because PKA does not affect arrestin recruitment^32^. *In vitro*, the V2Rpp and GRK2pp induce formation of the β-arrestin-1/clathrin complex, whereas the GRK6pp and V2Rpp promote the formation of the β-arrestin-1/SRC complex (Fig. 3c,d). By contrast, the PKA–phosphopeptide did not promote the formation of either the arrestin/clathrin complex or the arrestin/SRC complex (Fig. 3c,d). Taken together, these findings reveal that the GRK6-mediated receptor phosphorylation pattern specifically activates SRC, whereas the GRK2-mediated receptor phosphorylation pattern selectively recruits clathrin, thus facilitating receptor endocytosis (Table 1).

### Phospho-interaction patterns are deciphered by^19^F-NMR

**Figure 4:**
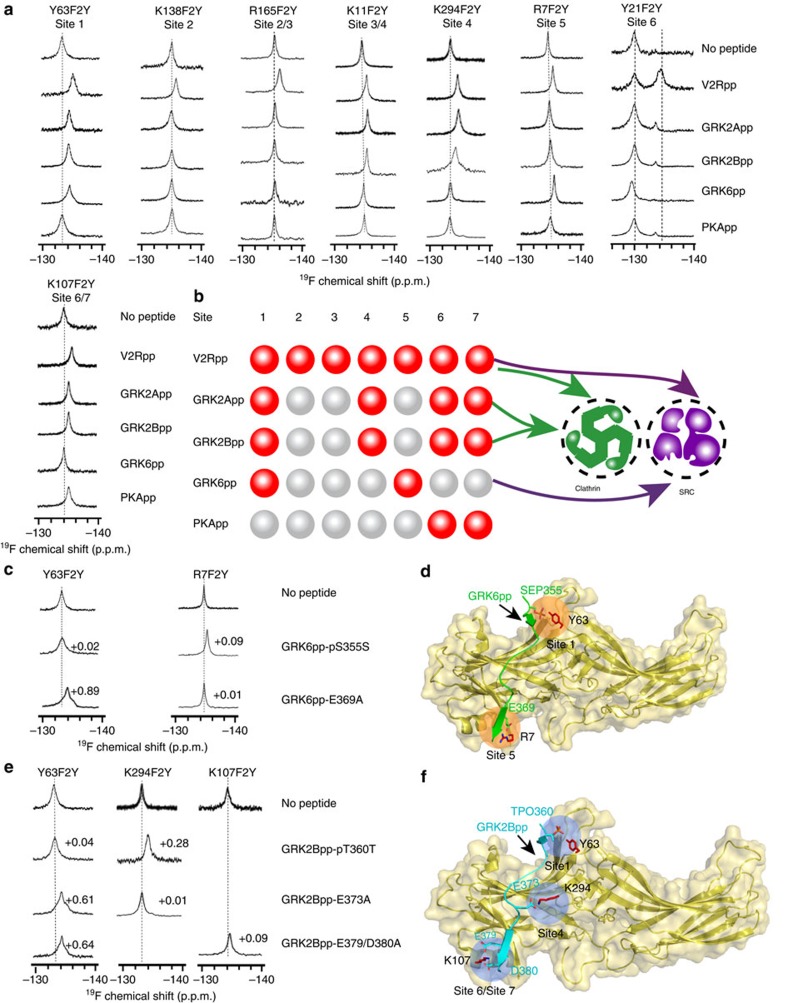
The phospho-interaction pattern of β-arrestin-1 was deciphered via the ^19^F-NMR spectra. (**a**) Effects of different phosphopeptide binding on the ^19^F-NMR spectra on β-arrestin-1-F2Y phospho-sensing probes. (**b**) Specific phospho-binding patterns revealed by ^19^F-NMR spectra that correlate to their biochemical and cellular properties. The red balls indicate that a phosphate or a negatively charged residue interacts with the specific phospho-binding site localized in the β-arrestin-1 N terminus. Grey balls indicate that no significant chemical shift increase (Δp.p.m.<0.05) was detected by the phospho-sensor around the specific site. The binding of the seven phosphate sites by V2Rpp promoted both clathrin and SRC binding. Two GRK2pps displayed similar phospho-binding patterns (1-4-6-7) and promoted clathrin binding but not SRC interaction. The phospho-binding pattern for GRK6pp is sites 1 and 5, which may correlate with its SRC function. (**c**) Effects of binding of GRK6pp mutants on the ^19^F-NMR spectra on β-arrestin-1-R7-F2Y and Y63-F2Y phospho-sensing probes. (**e**) Effects of binding of GRK2pp mutants on the ^19^F-NMR spectra on β-arrestin-1 phospho-sensing probes. (**d**,**f**) Structural representation of a model of specific interactions between the negative charged GRK6pp/GRK2pp residue and the residue localized in phospho-binding sites of β-arrestin-1. The model was generated by application of the PI-LZerD algorithm using V2Rpp/β-arrestin-1 complex structure (PDB: 4JQI) as a template and adjusted by results from ^19^F-NMR spectra.

V2Rpp, GRK2App, GRK2Bpp and GRK6pp have 11, 8, 9 and 4 phosphates or negatively charged residues, respectively. Specific phospho-binding patterns may correspond to distinct arrestin functions. We next used ^19^F-NMR spectroscopy of the F2Y phospho-sensors to detect the negative charge-sensing patterns of β-arrestin-1 after stimulation with specific phosphopeptides. All of the GRK–phosphopeptides, but not the PKA–phosphopeptides, induced an upfield chemical shift at Y63 (phosphate-binding site 1) (Fig. 4a). Specifically, GRK2App and GRK2Bpp caused upfield chemical shifts in ^19^F-NMR spectroscopy at positions K11, K294, K107 and Y21, which surround phosphate-binding sites 4, 6 and 7, whereas GRK6pp binding specifically induced an upfield chemical shift at R7, corresponding to phosphate-binding site 5 (Fig. 4a,b and Supplementary Table 2). A small amplitude of an upfield chemical shift was also observed at R165 after GRK6pp binding. However, this chemical shift was not due to the binding of a phosphate or a negative charge residue at phosphate binding site 2 or 3, as the binding of GRK6pp caused no significant chemical shift at either K11-F2Y, K138-F2Y or R62-F2Y (Fig. 4a and Supplementary Fig. 13 and Supplementary Table 2). Rather, the chemical shift at R165-F2Y after GRK6pp binding may reflect a conformational change at R165 or an interaction with R165 by a negatively charged residue with an unknown manner. A simplified phospho-interaction model of different phosphopeptides and their relations to arrestin functions are summarized in Fig. 4b.

To further characterize how these phosphopeptides interact with specific phosphate-binding sites of β-arrestin-1, we selectively mutated the negatively charged residues of these phosphopeptides to alanine and tested their effects on clathrin or SRC recruitment *in vitro*. Two mutations of GRK6pp and three mutations of GRK2pp affected phosphopeptide-induced β-arrestin-1/SRC or β-arrestin-1/clathrin interaction, respectively (Supplementary Figs 14 and 15). Importantly, the mutation of pS355 in GRK6pp selectively eliminated the upfield chemical shift at the Y63-F2Y position, whereas the mutation of E369A specifically abolished the upfield chemical shift at the R7-F2Y position after phosphopeptide binding (Fig. 4c). Therefore, the pS355 of GRK6pp interacts with phosphate-binding site 1 and E369 interacts with phosphate-binding site 5 in the phosphate-binding concave surface of β-arrestin-1 (Fig. 4d and Supplementary Table 7). Similarly, ^19^F-NMR spectroscopy with selective GRK2pp mutations indicated that pT360, E373 and E379/D380 specifically interact with phosphate-binding sites 1, 4 and 6/7, respectively (Fig. 4e,f).

### Phospho-barcodes induce structural changes in β-arrestin-1

**Figure 5:**
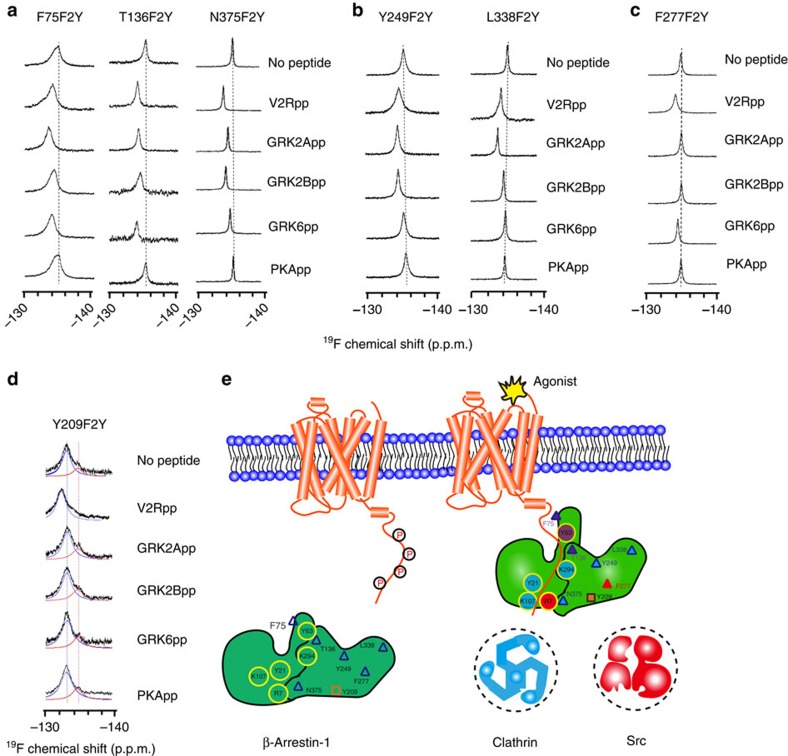
^19^F-NMR spectra revealed the different structural states of β-arrestin-1 in response to different phospho-binding patterns. (**a**) Common structural changes that occur after β-arrestin-1 activation induced by the binding of functional phosphopeptides. These results suggest the existence of common conformational states representing β-arrestin-1 activation after functional phosphopeptide binding. (**b**) Specific structural rearrangements after GRK2pp binding. (**c**) GRK6-encoded specific structural rearrangements at F277 position. (**d**) V2Rpp specifically stimulated conformational change at Y209 position. (**e**) Proposed models illustrating the receptor phospho-barcode-encoded distinct arrestin conformations that dictate specific downstream signalling. The phosphorylated C-tail of the receptor (in orange) extends into the cytoplasm. Interaction with the receptor phospho-C-tail induces a structural rearrangement of β-arrestin-1 that includes residues in the finger loop (such as F75) and the middle loop (such as T136); these residues (in purple) might participate in the receptor transmemberane core interaction. Different GRKs encode different conformations in the C-lobe of β-arrestin-1. GRK2 encodes specific conformations of Y249 and L338 (blue triangles), which are recognized by clathrin (in blue). GRK6 encodes distinct β-arrestin-1 conformations, such as the conformation of F277 (red triangle), which might be recognized by signalling proteins such as SRC (in red). Specific V2Rpp-induced conformational change is localized at Y209 (in orange).

The specific phosphopeptide/arrestin interaction pattern should trigger substantial arrestin conformational rearrangements. We next inspected the ^19^F-NMR spectrum of F2Y incorporation positions outside of the phosphopeptide interacting region. The binding of phosphate site 1 by GRK–phosphopeptides may provide the driving force for the repositioning of the finger loop and middle loop, which is a prelude to receptor core binding^2^,^33^. All of the GRK-phosphorylated peptides consistently induced downfield chemical shifts at residue 75 in the finger loop and at residue 136 in the middle loop (Fig. 5a and Supplementary Table 5). Therefore, except for PKApp, the common conformational changes that occur in the finger loop (residue 75) and middle loop (residue 136) regions that are induced in GRK–phosphopeptide binding (phosphate–Y63 interaction) might enable the interaction with the receptor cores for fully phosphorylated receptor/arrestin associations. Another important common structural feature of GRK–phosphopeptide binding is the downfield chemical shift at N375, which indicates the displacement of the C-tail of β-arrestin-1, despite the interaction of GRK2pp and GRK6pp with arrestin through different phosphate-binding sites in their C-terminal regions (sites 6 and 7 for GRK2pp and site 5 for GRK6pp; Figs 1b,c, 4b and 5a).

In particular, the interaction of GRK2pps (GRK2App and GRK2Bpp) with phosphate-binding site 4 enables the rotation of K294, releasing D290 and D297 from the polar core (Fig. 4a,b,e,f). The rearrangement of the polar core may lead to prominent twisting of the lariat loop, which facilitates the binding of the receptor transmembrane domain or clathrin. In addition, negatively charged residues or phosphates of both GRK2App and GRK2Bpp bind to phosphate sites 6 and 7, which disrupts the hydrophobic interactions between F388 and Y21/K107, resulting in the displacement of the C-tail and the conformational change at the amphipathic α-helix and the first two β-strands of β-arrestin-1. By contrast, although the binding of GRK6pp may also partially release the C terminus of β-arrestin-1, as it significantly increases the negative charge around R7 and causes a downfield shift at the Y21 position, it does not change the chemical shift at K294 or K107 (Fig. 4a–d and Supplementary Table 2). Therefore, selective GRK–phosphopeptide interaction patterns might result in distinct arrestin structural states. Accordingly, GRK2pp specifically induced a structural change at Y249 in the loop between β-strands XV and XVI, and L338-F2Y in the splice loop (Fig. 5b and Supplementary Table 5). By contrast, the GRK6pp, but not the GRK2pp or PKA–phosphopeptides, induced a specific structural change at F277-F2Y in the end of the lariat loop (Fig. 5c). Moreover, only V2Rpp induced a downfield chemical shift at the Y209-F2Y position in the ^19^F-NMR spectra, whereas all other tested phosphopeptides caused no significant effect (Fig. 5d and Supplementary Table 6). This conformational change at Y209 may be correlated with the binding of V2Rpp to phosphate-binding sites 2 or 3 of β-arrestin-1, which are the only two phosphate-binding sites that are not covered by GRK– or PKA–phosphopeptides (Fig. 4b). Taken together, these findings show that these phospho-barcode-induced structural rearrangements potentially enable the imparting of distinct functions of β-arrestin-1 through clathrin, SRC, ERK or other downstream effector proteins (Fig. 5e).

### Clathrin-induced structural alterations in β-arrestin-1

**Figure 6:**
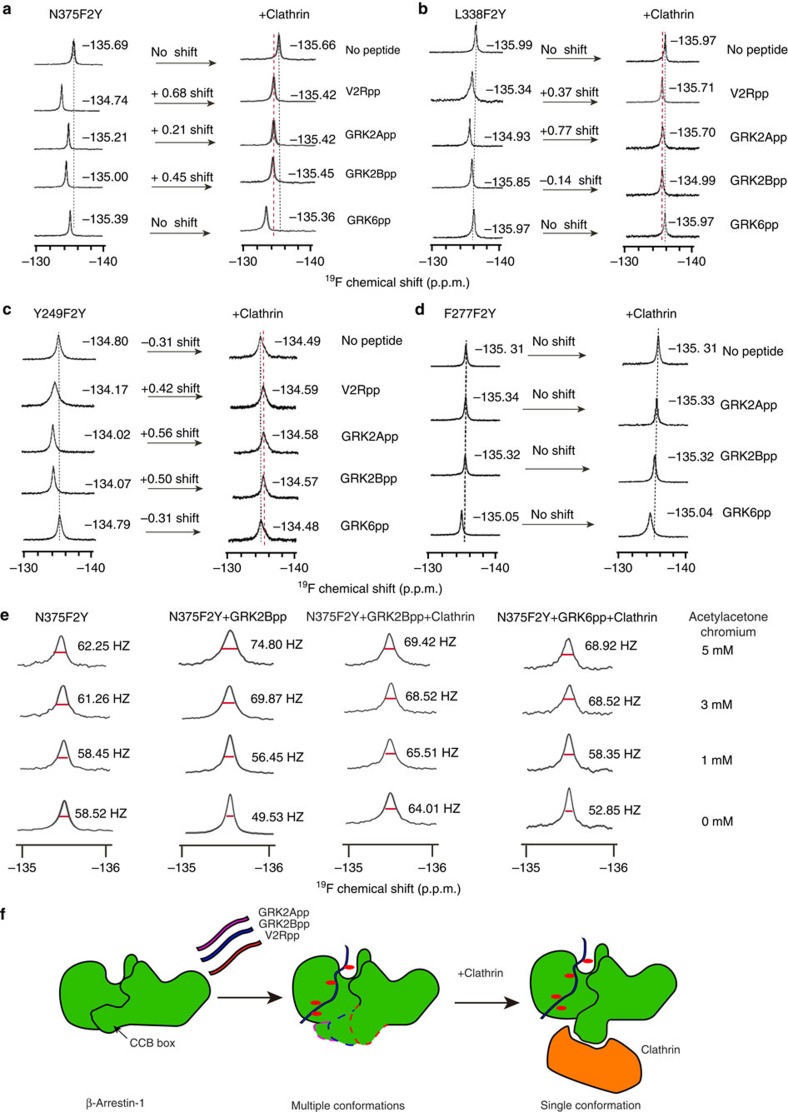
^19^F-NMR spectra revealed the structural alteration of β-arrestin-1 in response to the binding of Clathrin. Chemical shifts after phosphopeptide/β-arrestin-1/clathrin ternary complex formation at specific F2Y incorporation sites: at N375-F2Y close to the classic clathrin-binding box (**a**), at L388 in the splice loop (**b**) and at Y249-F2Y (**c**). (**d**) No significant changes (Δp.p.m.<0.05) were observed for the chemical shifts at F277-F2Y position in the ^19^F-NMR spectra after clathrin binding. (**e**) Paramagnetic titration experiments. As the concentration of the Cr increased, the full width at half maximum of the 1D-^19^F-NMR spectra at the F2Y-N375 position of the β-arrestin-1/GRK2Bpp complex significantly increased (Δp.p.m.=25 HZ), followed by the β-arrestin-1/GRK6 complex in the presence of clathrin (Δp.p.m.=16 HZ), and then the β-arrestin-1/GRK2Bpp/clathrin ternary complex (Δp.p.m.=5 HZ) and the β-arrestin-1 alone (Δp.p.m.=4 HZ), indicating a greater protective effect of Cr at the N375 position of the β-arrestin-1/GRK2Bpp complex in the absence of clathrin. (**f**) A cartoon illustration of the phospho-pattern-encoded structural rearrangements of β-arrestin-1 and its subsequent stabilization by clathrin binding. The red ball indicates the phosphate. Although the binding of GRK2App, GRK2Bpp and V2Rpp all caused a partial dislodging of the C terminus of β-arrestin-1, the resulting structural states induced by different phosphopeptides could be different. Clathrin binding stabilized all these phosphopeptide-occupied arrestins to a single state.

The GRK2-encoded arrestin conformation recruits clathrin and promotes receptor endocytosis. Clathrin-mediated phospho-receptor/arrestin complex trafficking is proposed to involve two distinct regions, the classic clathrin binding (CCB) box ^376^LIELD^380^ and the eight amino acid splice loop^34^. All F2Y mutations in the CCB box impaired the formation of the phosphopeptide/arrestin/clathrin complex (Supplementary Fig. 11). Thus, we selected L338-F2Y in the splice loop and N375-F2Y adjacent to the CCB box, to monitor clathrin-induced arrestin conformational changes. The binding of clathrin to the activated β-arrestin-1 in the presence of V2Rpp or GRK2pp led to a substantial chemical shift of the ^19^F-NMR signals at Y249-F2Y, L338-F2Y and N375-F2Y (Fig. 6a–c). Although GRK6pp also induced a structural change at N375-F2Y, clathrin binding caused no additional chemical shift (Fig. 6a). Moreover, the binding of clathrin to the phosphopeptide-occupied β-arrestin-1 did not affect the chemical shift at F277, a GRK6-specific structural site (Fig. 6d). These findings suggest that clathrin recognizes GRK2-encoded specific arrestin conformations of the splice loop, the CCB box and the loop between β-strands XV and XVI; however, clathrin does not recognize GRK6pp-encoded arrestin conformations. In particular, although V2Rpp and GRK2pp induce different amplitudes in the chemical shifts at L338-F2Y, Y249-F2Y and N375-F2Y, which suggests structural plasticity at these positions, the chemical shifts of the signals assigned to these positions in the ternary complexes of V2Rpp/arrestin/clathrin or GRK2pp/arrestin/clathrin exhibit no detectable differences (Fig. 6a–c). These results indicate that clathrin recognizes different phosphopeptide/arrestin intermediates and stabilizes the ternary complex to a common structural state at these positions.

We next assessed the effect of the paramagnetic relaxation agent chromium acetylacetone (Cr) on the ^19^F-NMR spectra of F2Y-N375 of β-arrestin-1 in the presence of clathrin and different GRKpp. The ^19^F-NMR spectra of F2Y-N375 revealed that the N375 position in the β-arrestin-1 alone or in the GRK2pp/β-arrestin-1/clathrin ternary complex is less susceptible to line broadening by Cr than the GRK2pp/β-arrestin-1 complex or the GRK6pp/β-arrestin-1 complex plus clathrin, indicating that F2Y-N375 is less readily solvent accessible in β-arrestin-1 alone or the GRK2pp/β-arrestin-1/clathrin complex (Fig. 6e and Supplementary Fig. 16). Therefore, a model was built in which the C-terminal region of β-arrestin-1 was dislodged after GRKpp binding, which was protected by clathrin in the GRK2pp/β-arrestin-1 complex but not in the GRK6pp/β-arrestin-1 complex (Fig. 6f).

### Phospho-decision mechanisms of β-arrestin-1 functions

**Figure 7:**
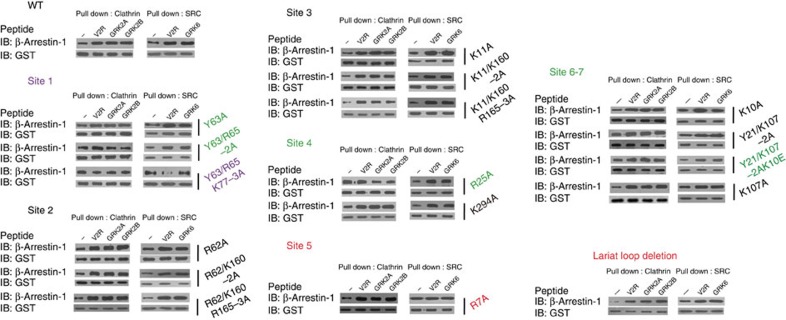
Specific phospho-sensors located in the N terminus of β-arrestin-1 determine its selective interaction with clathrin or SRC. Systematic Ala scanning mutations covering all seven phosphate-binding sites of β-arrestin-1 were examined for their effects on phosphopeptide-induced clathrin or SRC recruitment. The effects of phosphopeptide binding on β-arrestin-1/clathrin complex formation (left panel) and β-arrestin-1/SRC3D complex formation (right panel) were examined with a GST pull-down assay. Representative western blottings from at least three independent experiments for each assay are shown. The K10/Y21/K107-3A mutant was not expressed and therefore changed according to the combination of K21/K107-2A-K10E. Destroying phosphate-binding site 1 by Y63/R65/K77-3A abolished both phosphopeptide-induced clathrin and SRC recruitment (shown in magnate). Mutation in site 4 by R25A eliminated, and the mutation at site 6/7 by Y21/K107-2A/K10E, decreased phosphopeptide-induced clathrin binding but had no effect on SRC interaction (in green). Conversely, the mutation at site 5 by R7A and the partial deletion of the lariat loop decreased the SRC interaction but had no effect on phosphopeptide-induced clathrin binding (in red). All other mutations had no significant effects on phosphopeptide-induced SRC or clathrin recruitment.

To further characterize the functional outcomes of the specific phospho-interaction pattern and the accompanying structural rearrangements of β-arrestin-1, we generated systematic β-arrestin-1 mutants that covered all seven V2R phosphate-binding sites and a partial lariat loop deletion construct. Mutations of all residues in phosphate-binding sites 2 or 3 had no significant effects on clathrin or SRC binding. By contrast, destroying phosphate-binding site 1 by Y63/R65/K77-3A, which is essential for the finger loop rearrangement, eliminated both clathrin and SRC binding (Fig. 7 and Supplementary Fig. 17). More interestingly, the GRK2pp-stimulated clathrin binding was significantly reduced by mutations of phosphate-binding sites 6 and 7 (Y21A/K107A/K10E) and was entirely eliminated by a single mutation of R25A in phosphate-binding site 4. However, these mutations have no significant effects on the GRK6pp-induced SRC/β-arrestin-1 interaction. Furthermore, the interruption of the GRK6pp-specific interacting site by R7A significantly reduced the phosphopeptide-induced SRC binding but had no noticeable effect on clathrin binding. Accordingly, partial deletion of the lariat loop close to the F277 position, which only exhibits structural rearrangement after GRK6pp binding, resulted in a decreased phosphopeptide-induced SRC interaction (Fig. 7 and Supplementary Fig. 17). These results indicated that phosphate-binding sites 4, 6 and 7 govern phosphopeptide-induced clathrin binding, whereas phosphate-binding site 5 defines the SRC interaction. The binding of phosphopeptides to phosphate-binding site 1 primes both SRC and clathrin recruitment.

**Figure 8:**
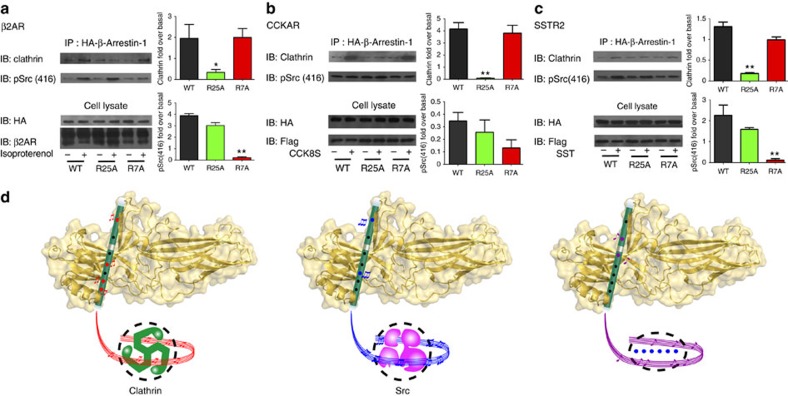
Phospho-pattern-selective mechanisms of specific arrestin functions are common for at least a subset of GPCRs. (**a**) The phospho-binding pattern of β-arrestin-1 determines selective arrestin functions downstream of β2AR in cells. HEK293 cells were co-transfected with Flag-β2AR and wild-type HA-β-arrestin-1 or specific phosphate-binding site mutants (R25A for site 4 and R7A for site 5), then stimulated with 10 μM isoproterenol (ISO) for 15 min. The β-arrestin-1 was immunoprecipitated by HA-antibody-conjugated agarose and the formation of the β-arrestin-1/clathrin or β-arrestin-1/SRC was detected by specific clathrin or SRC antibodies. R25A or R7A selectively disrupted the ISO-induced β-arrestin-1/clathrin complex formation or the ISO-induced β-arrestin-1/SRC complex formation, respectively. (**b**,**c**) The phospho-binding pattern of β-arrestin-1 determines selective arrestin interaction with clathrin or SRC downstream of somatostatin receptor 2 (SSTR2, Fig. 4c) and cholecystokinin A receptor (CCKAR, Fig. 4d). The Flag-CCKAR or Flag-SSTR2 and HA-β-arrestin-1 plasmids were co-transfected in HEK293 cells. The cells were starved and then stimulated with 1 μM somatostatin (**c**) or 2 μM CCK-8s (**d**) for 15 min, respectively. The somatostatin promoted both clathrin and SRC recruitment for β-arrestin-1, whereas the CCK-8s only promoted β-arrestin-1/clathrin interaction. The site 4 mutation R25A selectively disrupted β-arrestin-1/clathrin interaction after somatostatin or CCK-8s application and the site 5 mutation R7A impaired the β-arrestin-1/SRC complex formation after somatostatin stimulation. (**a**–**c**) Representative western blottings from at least three experiments are shown on the left and the statistics from at least three independent experiments are shown on the right. *P<0.05, **P<0.01; the effects of mutants were compared with the wild type. (**d**) A proposed model for arrestin signalling encoded by the phospho-C-tail of the GPCRs. The phosphate-binding sites arranged in the N terminus of arrestin look similar to the holes in a flute. Left: the pattern 1-4-6-7 directed a specific arrestin conformation for clathrin signalling. Middle, the pattern 1-5 directed specific SRC signalling. Right: other combinations may direct other GPCR signalling.

Consistent with these biochemical results, the interruption of phosphate-binding site 4 by R25A selectively impaired β-arrestin-1/clathrin interaction downstream of β2AR activation, whereas destroying phosphate-binding site 5 specifically eliminated ISO-induced β-arrestin-1/SRC association (Fig. 8a). A similar result for arrestin/partner interactions by specific phospho-interaction patterns was also observed in CCKAR for clathrin and in SSTR2 for clathrin and SRC (Fig. 8b,c). Therefore, this newly identified phospho-pattern decision mechanism also underlies at least a subset of GPCRs.

## Discussion

Understanding the coding mechanisms and structural basis of arrestin-mediated GPCR signalling is currently a central issue in receptor biology that may have important implications for new drug development. In contrast to the tremendous effort focused on understanding the functional significance of the conformational heterogeneity of receptors^1^,^35^,^36^,^37^,^38^,^39^, the importance of the structural plasticity of arrestin has only just begun to be analysed^11^,^40^,^41^,^42^. Recent studies have suggested that ligand-specific receptor conformation is correlated with, and that the C-terminal phosphorylation pattern defines, specific arrestin conformations that dictate selective signalling pathways^1^,^5^,^13^. The marked conformational rearrangement of arrestin in the crystal structure of the β-arrestin-1/V2Rpp/Fab30 complex provides considerable insight into the structural properties of arrestin and further supports the idea that specific conformations underlie selective arrestin functions^2^. However, this crystal structure only presents a static picture for a single activated arrestin conformation. Certain important structural features that are highly relevant to arrestin functions, such as various loops and the final 50 amino acids at the C terminus of arrestin, are absent from the structure of the β-arrestin-1/V2Rpp complex because of poor electron densities^11^. Moreover, the ways in which the specific structural states of arrestins are defined by specific phosphorylated cytoplasmic regions of a receptor or by ligand-occupied receptor conformations remain uncertain and correlations between specific arrestin conformations and distinct arrestin functions have not been established. In addition, the flexible nature of the arrestin structure has hampered crystallographic analysis in the absence of stabilization by conformation-specific antibodies. In this context, we have developed a series of F2Y-based structural sensors in β-arrestin-1 through unnatural amino acid incorporation (Fig. 1a–c). These sensors enabled us to use ^19^F-NMR spectroscopy to inspect phospho-interaction patterns and residue-specific structural information in β-arrestin in different active states (Fig. 2a,e).

Although there is no apparent phospho-sequence identity between GRK2App and GRK2Bpp, ^19^F-NMR spectra of F2Y incorporated into the phosphate-binding sites of β-arrestin-1 indicated that these phosphopeptides interact with β-arrestin-1 through identical phosphate-binding sites (1-4-6-7) (Table 1 and Fig. 4b). In contrast, a different phospho-interaction pattern was observed for the interaction between GRK6pp and β-arrestin-1 (1-5) (Fig. 4b). Analyses of ^19^F-NMR spectra allowed us to reveal structural changes common to all functional phosphopeptide binding. These common changes include alterations to residue F75 in the finger loop, T136 in the middle loop and N375 in the C-terminal region (Fig. 5a). Recent EM and crystallographic studies have suggested that the finger loop of arrestins is inserted into transmembrane (TM) helices and intracellular loops of receptors after receptor/arrestin complex formation^2^,^43^. Therefore, structural changes at the finger loop and middle loop may be an important common mechanism in promoting the formation of tight receptor/arrestin complexes after arrestin reads specific receptor phosphorylation signals. Apart from the polar core of the central portion of the β-arrestin-1 protein, the C-terminal tail of β-arrestin-1 (encompassing N375) runs in the opposite direction to the finger loop and the middle loop. Two potentially different modes of binding between β-arrestin-1 and clathrin in a non-stimulated state have been proposed by the complex structure of clathrin and β-arrestin-1 (ref. 34). However, the interaction mode between active arrestin and clathrin has not been defined. In the present study, relaxation experiments at position N375 with different conditions enabled us to demonstrate that the C-terminal region of β-arrestin-1 is protected in the GRK2Bpp/β-arrestin-1/clathrin ternary complex (Fig. 6e,f). Thus, our results suggest that the receptor and clathrin interact with β-arrestin-1 in opposite directions to form a sandwich-like structure during receptor internalization.

More importantly, ^19^F-NMR spectra at other F2Y-incorporated sites provide direct biophysical evidence for the presence of at least three unique active β-arrestin-1 structural states corresponding to different phospho-binding patterns. The change at F277 is specific to GRK6pp (phospho pattern 1-5), the changes at the Y249 and L338 positions are specific to GRK2pp (phospho pattern 1-4-6-7) and the change at the Y209 position is unique to V2Rpp (phospho pattern-1-2-3-4-5-6-7) (Fig. 5b–d). By maintaining or disrupting the polar core of β-arrestin-1, specific interactions at phosphate-binding site 4 may play a causative role in discriminating among distinct arrestin conformations induced by the binding of different GRKpps. Accordingly, the change in ^19^F-NMR spectra at GRK2-specific sites but not GRK6-specific sites after clathrin binding, in combination with GST pull-down and cellular studies, further confirms the coupling of distinct arrestin structural states to selective arrestin functions (Figs 6a–b, 7 and 8a–c). Taken together, the experiments in our study elucidate a key step in the working mechanism of GPCRs; in particular, the phospho-barcode of the receptor is harboured in the phosphate-binding concave surface of β-arrestin-1 and translated by arrestin into specific structural states that direct distinct arrestin-mediated functions (Fig. 8d).

In addition to revealing the phospho-coding mechanism of the specific functions of β-arrestin-1, ^19^F-NMR spectroscopy enabled us to probe for dynamic and subtle conformational changes at specific F2Y-incorporated sites. For example, the ^19^F-NMR spectra reveal the presence of both slow and fast exchanges between different conformations at the Y21-F2Y and Y209-F2Y positions (Fig. 2a,b,e). Specifically, Y21-F2Y consists of two components after V2Rpp binding, indicating the presence of two independent equilibria between locally different conformations in the presence of the V2Rpp. Increasing the V2Rpp concentration lead to the increased size of the upfield-shifted peak of F2Y-Y21 at position −135.22 (Fig. 2b and Supplementary Table 3). Moreover, the dose–response curve of V2Rpp-stimulated clathrin recruitment was directly correlated with the increased peak area of F2Y-Y21 at the −135.22 position, suggesting that the equilibrium at −135.22 represents an ‘active' arrestin conformation (Supplementary Fig. 6). It is worth noting that Y21 forms a hydrophobic interaction with the F388 of the C-tail, which is important in maintaining the ‘three elements interaction' in the ‘inactive' arrestin structure (Supplementary Fig. 5c). In the previous model, binding of the phosphorylated receptor C-tail removed the proximal C-terminal region of β-arrestin. Consistent with this hypothesis, the paramagnetic relaxation experiment at Y21 suggested that V2Rpp binding induced greater solvent accessibility at the −135.22 equilibrium position (Supplementary Fig. 5). Similar to the binding of V2Rpp, the binding of GRK2App and GRK2Bpp to β-arrestin-1 induced two independent equilibria at the Y21-F2Y position (Fig. 4a). The population of active peaks (at the −135.05 position) induced by GRK2pp binding was smaller than that caused by V2Rpp binding, consistent with the lower efficacy of GRK2pp in promoting clathrin recruitment than that of V2Rpp (Fig. 3d). Interestingly, even in the saturated V2Rpp concentration, half of the F2Y-Y21 of β-arrestin still presented as an ‘inactive' structural state (peak at −130.18) (Fig. 2b). This result indicated the possibility that not all β-arrestin-1 proteins are activated during the signal transduction, or that the complete activation of β-arrestin-1 requires other activating elements, such as binding of the receptor transmembrane core.

In contrast to visual arrestin that forms oligomers in physiological conditions, the full-length β-arrestin-1 is mainly a monomer in solution^44^. However, a truncated version of β-arrestin-1 (1–382) exists as mixtures of monomer and dimer, which implies that the oligomerization state of β-arrestin-1 might be regulated under appropriate physiological conditions^45^. Among the 17 F2Y-incorporated positions of β-arrestin-1 in the present study, the Y249 and L338 positions are in the vicinity of the dimer interface of the truncated β-arrestin-1 (1–382) (Supplementary Fig. 18). Although our NMR experiments used full-length β-arrestin-1, which is unlikely to form dimers during data collection, the observed ^19^F-NMR changes at these positions may reflect the fact that the binding of phosphopeptide or clathrin can change the oligomerization propensity of β-arrestin-1 under specific cellular conditions. Recently, the functional importance of homodimerization and heterodimerization has been extensively studied at the receptor level^38^,^46^,^47^. Although the functional relevance of arrestin oligomers remains relatively unknown, oligomeric forms of visual arrestins have been observed under physiological conditions and studies have found that β-arrestins concentrate in specific subcellular localizations to allow for dimer formation after receptor activation^48^,^49^,^50^. The formation of β-arrestin oligomers might provide a platform for the binding of receptor dimers or other downstream effectors, including Mdm2 or other proteins^50^, leading to the formation of higher-order functional complexes.

In conclusion, we have clarified a longstanding question regarding the receptor phospho-coding mechanism that dictates selective β-arrestin-1 structural features and signalling. The phosphate-binding sites along the N terminus of β-arrestin-1 are arranged in a shape that is similar to the holes in a flute and arrestin moves differently according to the instructions of the phospho-receptor ‘fingers'. Whereas a receptor-encoded 1-4-6-7 pattern determines the specific β-arrestin-1 conformation for clathrin recruitment, a different receptor ‘tune,' 1-5, provides the SRC signalling order (Fig. 8d). Moreover, the N terminus of β-arrestin-1 harbours three potential additional phosphate-binding sites (A1–A3; Supplementary Fig. 19). The combination of all phosphate-binding sites in a single arrestin allows the expression of more than 1,000 patterns (2^10^–1=1,023) that allow it, in theory, to produce a plethora of arrestin conformations, facilitating numerous downstream protein interactions^51^. Most of the phosphorylation binding sites of β-arrestin-1 consist of conserved residues, indicating their common roles in the recognition of phospho-barcodes in different arrestin members and across different species (Supplementary Figs 2 and 3). Activation of the same ‘G subtype protein-coupled GPCR' can have distinct functions in cells. Although some functional differences are attributed to the duration and concentration of increased levels of second messengers^52^, many functional differences in receptors may result from distinct arrestin functions^53^,^54^.The revealed ability of arrestin to read and translate various phosphorylation patterns through its phosphate-binding concave surface contributes to the diverse functions of more than 800 GPCRs present in the human genome. The phospho-receptor C-tail may work together with different ligand-induced receptor conformations and orchestrate the breadth of GPCR signalling complexity.

## Methods

### Reagents

The monoclonal anti-phospho-SRC (pTyr-416, 2101), anti-GST (2622) and anti-YFP (2555) antibodies were from Cell Signaling. The anti-c-SRC (60315-1-Ig), anti-GRK2 (13990-1-AP) and anti-GRK6 (11439-1-AP) antibodies were from Proteintech (Chicago, USA). The monoclonal anti-Flag M2 antibody (F3165) were purchased from Sigma. The anti-β2AR(sc-569), anti-haemagglutinin (HA)(sc-7392) and anti-Clathrin (sc-12734) antibodies were from Santa Cruz. Most of the antibodies are used at a 1:1,000 dilution, except for anti-clathrin antibody at 1:500. The HA and Flag beads were purchased from Sigma. Glutathione-Sepharose 4B and Ni-NTA Agarose were from Amersham Pharmacia Biotech. V2Rpp and GRK2App were synthesized by the Tufts University core facility (Boston, USA). GRK2Bpp, GRK6pp and PKApp were synthesized by China Peptides Co., Ltd. (Shanghai, China). The monoclonal anti-β-arrestin-1 antibody was a generous gift from Dr Lefkowitz at Duke University. All of the other reagents were from Sigma.

### Constructs

The pcDNA3.0-Flag-β2AR (Flag-β2AR), β-arrestin-1-YFP, pcDNA3.1-Luc-β-arrestin-2-YFP and pCDNA3.1-Clathrin constructs were generous gifts from Dr Lefkowitz at Duke University^21^,^32^. The chicken c-SRC (residues 87–531, containing SH3, SH2 and catalytic domains) was a generous gift from Dr Zhong-yin Zhang at Indiana University and John Kuriyan at UC Berkeley^55^,^56^. For F2Y-incorporated protein expression, the pEVOL-F2YRS plasmid used has been described previously^19^. To monitor the β-arrestin-1 conformational change, the BRET sensor for β-arrestin-1 (pcDNA3.1-Luc-β-arrestin-1-YFP) was created by in-Fusion of the β-arrestin-1-YFP plasmid with the pcDNA3.1-Luc-β-arrestin-2-YFP construct^57^,^58^. For *E. coli* expression, the full-length wild-type complementary DNAs of bovine β-arrestin-1 was subcloned into the NdeI/XhoI sites of the pET22b vector with the C-terminal His tag. DNA encoding residues 1–494 of human clathrin and chicken c-SRC (residues 87–531, containing SH3, SH2 and catalytic domains) were sub-cloned into a PGEX-6P1 expression vector with an N-terminal GST tag for *E. coli* expression and GST pull-down assays. The β-arrestin-1 mutations Y21TAG, Y63TAG, F75TAG, T136TAG, Y249TAG, F277TAG, L338TAG, L379TAG, F75Y, T136Y, F277Y, L338Y and L379Y were generated using the Quikchange mutagenesis kit (Stratagene). All of the constructs and mutations were verified by DNA sequencing.

### Cell culture, siRNA and transfection

HEK 293 cells were attained from ATCC and cultured at 37 °C and 5% CO_2_ in DMEM containing 10% (v/v) fetal bovine serum and penicillin–streptomycin (5,000 IU l^−1^). GRK2 or GRK6 expression was silenced in HEK 293 cells using a specific small interfering RNA (siRNA) duplex. The GRK2 target sequences were 5′-AAGAAGUACGAGAAGCUGGAG-3′ and the GRK6 target sequences were 5′-AACAGUAGGUUUGUAGUGAGC-3′ as previously described^5^,^59^,^60^. Cells were transfected with GRK siRNA, control siRNA (a non-targeting 25-nt siRNA) or other plasmids by Lipofectamine 2000 according to the manufacturer's instructions.

### Western blotting

To examine the roles of GRK2 and GRK6 in β2-AR-mediated SRC activation, selective siRNA towards specific GRK and Flag-β2AR plasmids were co-transfected into HEK-293 cells. After 24 h of transfection, the cells were starved for 4 h and then stimulated with ISO (10 μM) at the indicated times. Subsequently, the cells were washed three times with cold PBS and then collected in cold lysis buffer (50 mM Tris pH 7.5, 150 mMNaCl, 1% Triton X-100, 1 mM EGTA, 1 mM Na_3_VO_4_, 50 mMNaF, 0.25% (m/v) sodium deoxycholate, 10% (v/v) glycerol, a protease inhibitor cocktail tablet and 5 mM IAA). The cell lysates were centrifuged for 15 min after 30 min of end-to-end rotation at 4 °C. Lysate protein concentrations were determined using the Bradford protein assay. Equal amounts of lysate proteins were subjected to SDS–PAGE and western blotting. SRC-416 phosphorylation was detected using a specific antibody. The original pictures for uncropped gels are shown in Supplementary Figs 20 and 21.

### Co-immunoprecipitation

To examine the effects of β-arrestin-1 mutants on ISO-induced β2AR/β-arrestin-1 complex formation, the co-immunoprecipitation experiment was performed^61^,^62^. In detail, HEK293 cells were co-transfected with Flag-β2AR and β-arrestin-1-YFP mutants. Twenty-four hours after transfection, the cells were starved for 4 h and then stimulated with ISO (10 μM) for 10 min. The cell lysates were subjected to immunoprecipitation using anti-FLAG beads incubated overnight at 4 °C. Immune complexes were analysed by western blotting with specific antibodies. The associated β-arrestin 1-YFP was detected using an anti-YFP antibody.

To examine the effects of β-arrestin-1 mutants on ISO, CCK-8s or somatostatin induced clathrin/β-arrestin-1 or SRC/β-arrestin-1 complex formation downstream of β2AR, CCKAR and SSTR2; HEK293 cells were co-transfected with Flag-β2AR/HA-β-arrestin-1, Flag-CCKAR/HA-β-arrestin-1 or Flag-SSTR2/HA-β-arrestin-1, respectively. Different HA-β-arrestin-1 mutations were used to detect the contribution of the phosphate-binding sites of β-arrestin-1 during the arrestin/clathrin or arrestin/SRC complex formatin. Forty-eight hours after transfection, the cells were starved for 8 h and then stimulated with ISO (10 μM for activation of β2AR), CCK-8s (2 μM for activation of CCKAR) or somatostatin (1 μM for activation of SSTR2) for 15 min. The cell lysates were subjected to immunoprecipitation using anti-HA beads incubated overnight at 4 °C. Immune complexes were analysed by western blotting with specific antibody towards clathrin or pSRC416, respectively.Representative western blots from at least three experiments are shown in Fig. 8.

### Confocal microscopy

Confocal microscopy experiment was performed^30^. The plasmids encoding the β-arrestin-1 BRET biosensor (Luc-β-arrestin-1-YFP) or β-arrestin-1-YFP mutants were transiently co-transfected with Flag-β2AR in HEK-293 cells. Twenty-four hours after transfection, the cells were plated on fibronectin-coated, 35-mm, glass-bottom plates. After 4 h of starvation, the cells were stimulated with ISO (10 μM) for 10 min. The translocation of β-arrestin-1 was monitored by VT-Infinity Confocal Imaging System.

### BRET assay

The plasmids Luc-β-arrestin-1-YFP, Flag-β2AR and different siRNAs were transiently co-transfected in HEK-293 cells. Twenty-four hours after transfection, HEK-293 cells were distributed in fibronectin-coated 96-well microplates. Before the BRET assay, cells were washed three times with PBS and then cells were incubated with coelenterazine h (final concentration, 5 μM) for 5 min. Subsequently, the cells were stimulated with 10 μM ISO for 10 min and light emission was detected (460–500 nm for Luc and 510–550 nm for yellow fluorescent protein (YFP)) using a multilabel reader (Mithras Luria Bertani 940; Berthold Technologies). The BRET signal was determined as the ratio of the light emitted by YFP and the light emitted by Luc.

### Expression and purification of native β-arrestin-1

The plasmid pET22b-β-arrestin 1 was transformed into BL21 *E. coli*. The transformed BL21 cells were cultured and induced with 0.3 mM isopropyl-β-D-thiogalactoside (IPTG) at an OD600 nm of 0.8. After growing overnight at 25 °C, the cells were harvested, re-suspended in lysis buffer (20 mM Tris-HCl pH 8.0, 150 mM NaCl) and lysed by sonication. The bacterial lysate was next centrifuged and the supernatant was purified by Ni-NTA affinity chromatography and Superdex 200 according to the manufacturer's instructions (GE Healthcare).

### Expression and purification of F2Y-incorporated proteins

For the expression of β-arrestin-1 F2Y proteins, pEVOL-F2YRS was co-transformed with different pET22b-β-arrestin-1 TAG mutations into BL21 (DE3). A single colony was grown overnight at 37 °C in Luria Bertani (LB) medium. Five litres of the transformed cells were then induced with 0.3 mM IPTG and 0.02% L-arabinose at an OD600 nm of 1.0 in the presence of 0.5 mM F2Y. After growing overnight at 25 °C, the cells were harvested and resuspended in buffer containing 20 mM Tris-HCl pH 8.0 and 150 mM NaCl. The bacterial lysate was centrifuged and the supernatant was purified by Ni-NTA affinity chromatography and gel filtration chromatography.

### Trypsin digestion and MS/MS analysis

The β-arrestin-1-F2Y protein was subjected to electrophoresis and the protein band was cut into small plugs and washed twice in 200 μl distilled water for 10 min. The gel bands were dehydrated in 100% acetonitrile for 10 min and dried in a Speedvac (Labconco) for ∼15 min. Disulfide bonds were reduced by adding 10 μl of 100 mM dithiothreitol (DTT) and subsequently alkylated by 40 mM IAA, 25 mM NH_4_HCO_3_ for 45 min at room temperature in the dark. The sample was then mixed with trypsin by a ratio of 100:1 in Tris buffer and digested at 37 °C for 12 h. Digestion was stopped by adding formic acid to 1% final concentration. Digested samples were purified and desalted, and re-dissolved in 30 μl 50% CH3CN/0.1% CF3COOH buffer before MS/MS analysis.

LC-MS/MS analysis was performed using a Thermo Finnigan LTQ linear ion trap mass spectrometer in line with a Thermo Finnigan Surveyor MS Pump Plus HPLC system. The peptides generated by trypsin digestion were loaded onto a trap column (300SB-C18, 5 × 0.3 mm, 5 μm particle) (Agilent Technologies, Santa Clara, CA), which was connected through a zero dead volume union to the self-packed analytical column (C18, 100 μm i.d × 100 mm, 3 μm particle) (SunChrom, Germany). The peptides were then eluted over a gradient (0–45% B in 55 min, 45–100% B in 10 min, where B=80% Acetonitrile, 0.1% formic acid) at a flow rate of 500 nl min^−1^ and introduced online into the linear ion trap mass spectrometer (ThermoFisher Corporation, San Jose, CA) using nano electrospray ionization. MS data were analysed by Bioworks 3.2 software.

### Expression and purification of clathrin and SRC 3D proteins

GST-tagged Clathrin or SRC 3D Protein was expressed in BL21 *E. coli*. In brief, 5 l of GST-tagged Clathrin or SRC 3D-transformed *E. coli* was cultured, induced by 0.3 mM IPTG and pelleted by centrifugation. The cell pellets were resuspended in 50 ml of a GST buffer containing 25 mM Tris-HCl at pH 8.0, 150 mM NaCl, 0.5% Triton X-100, 5% glycerol, 2 mM EDTA and 1 mM DTT. The bacterial lysate was centrifuged and the supernatant was collected and incubated with 2 ml of glutathione-Sepharose 4B for 2 h. The beads were washed three times and the bound GST-Clathrin protein or GST-SRC protein was eluted with 10 mM GSH for GST pull-down experiments. To acquire clathrin for phospho-peptide/β-arrestin-1/clathrin complex formation, GST-Clathrin bound to GST beads was mixed with HRV-3C protease for 12 h at 4 °C. The cleaved protein was concentrated to 500 μl and subjected to gel filtration using a Superdex 200 column (GE Healthcare) before being added to the phosphopeptide/β-arrestin-1 complex.

### Clathrin/SRC-binding assay

Binding of clathrin or SRC to β-arrestin-1 was performed as previously described^7^,^63^,^64^. In detail, 300 nM wild-type β-arrestin-1 from F2Y incorporated proteins were first mixed with specific phosphopeptides and incubated in binding buffer (20 mM Tris-HCl, pH 8.0, 150 mM NaCl, 2 mM EDTA, 1 mM DTT) at room temperature for 30 min. After the incubation, 10 μM GST-clathrin or GST-SRC protein was added and incubated for another 1 h at room temperature. Subsequently, 10 μl of GST beads were added to the mixture. After 2 h of end-to-end rotation at 4 °C, the beads were collected and washed six times with cell lysis buffer. After removing the supernatant in the final wash, the samples were re-suspended in 2 × SDS loading buffer and boiled for 10 min. The arrestin/clathrin or arrestin/SRC complexes were analysed by western blotting. For each experiment, a western blot representative of at least three independent experiments is shown in Figures.

### NMR experiments

To detect phosphopeptide-induced arrestin conformational changes, 50 μM β-arrestin-1 F2Y proteins were mixed with or without a threefold molar ratio of phosphopeptides (V2Rpp, GRK2App, GRK2Bpp, GRK6pp or PKApp) and incubated in binding buffer (20 mM Tris-HCl pH 8.0, 150 mM NaCl, 10% D_2_O) with end-to-end rotation at room temperature for 30 min. To detect the arrestin conformational change after clathrin binding, an equal molar of clathrin was added to the arrestin or arrestin/phosphopeptide complex and incubated for another 30 min. The protein samples were then subjected to ^19^F-NMR experiments.

For the titration experiment, 50 μM β-arrestin-1-Y21-F2Y or β-arrestin-1-Y63-F2Y proteins were mixed at different ratios (0.3:1, 1:1, 3:1 and 9:1) of the V2Rpp and incubated in binding buffer (20 mM Tris-HCl pH 8.0, 150 mM NaCl, 10% D_2_O) at room temperature for 30 min. The proteins were then subjected to ^19^F NMR experiments.

All NMR data were collected using an Agilent OD2 600 spectrometer fitted with a 5-mm broad band probe. The ^19^F 90° pulse lengths were 9.9 μs and the spectra were typically obtained using 15,000 scans and a recovery delay of 1 s. Data were processed using 10-Hz Lorentzian line broadening and were referenced to the internal TFA standard (−76.5 p.p.m.). All of the spectra were recorded at 25 °C.

## Additional information

**How to cite this article:** Yang, F. *et al.* Phospho-selective mechanisms of arrestin conformations and functions revealed by unnatural amino acid incorporation and ^19^F-NMR. *Nat. Commun.* 6:8202 doi: 10.1038/ncomms9202 (2015).

## Supplementary Material

Supplementary Information Supplementary Figures 1-21 and Supplementary Tables 1-7

## Figures and Tables

**Table 1:**
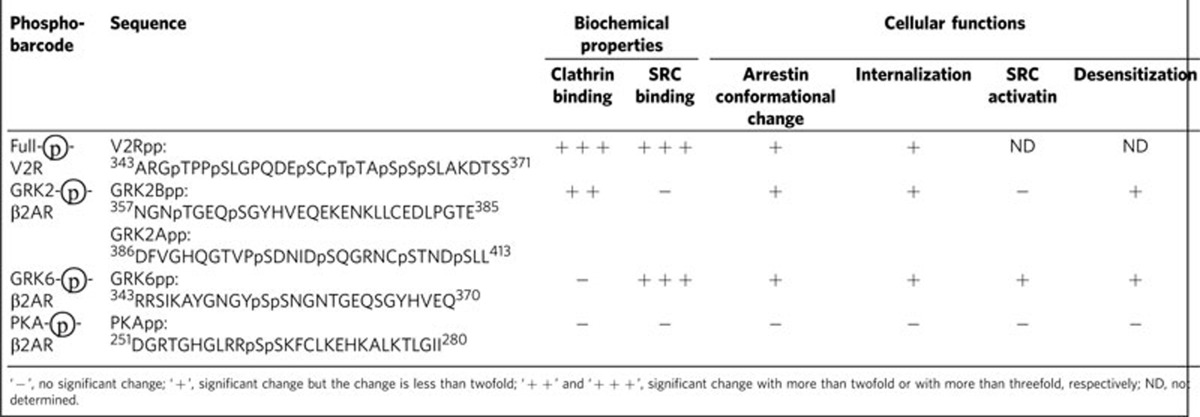
Summary of the signalling and biochemical properties of receptor phosphorylation barcodes encoded by various kinases.
