# Supplementary material for: Phospho-selective mechanisms of arrestin conformations and functions revealed by unnatural amino acid incorporation and 19F-NMR
Source: Nat Commun. 2015 Sep 8;6:8202. doi: 10.1038/ncomms9202 (PMC4569848; doi:10.1038/ncomms9202)
Supplement: Supplementary Information — Supplementary Figures 1-21 and Supplementary Tables 1-7 [file ncomms9202-s1.pdf]

Supplementary Figure 1.

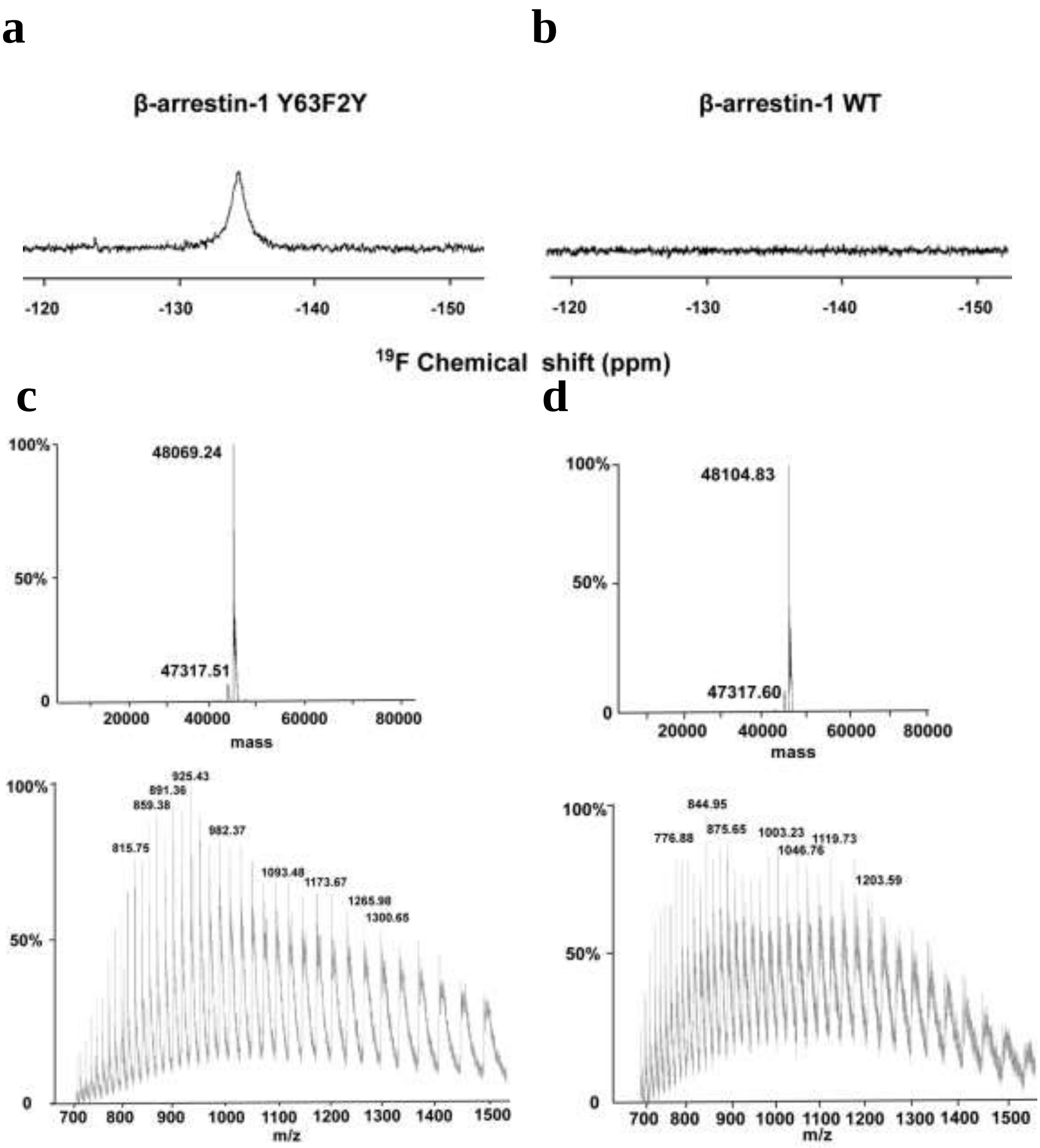

Supplementary Figure 1. ^{19}F-NMR spectrum for the Y63-F2Y mutant of β-arrestin-1 vs. wild-type β-arrestin-1 (WT).

The β-arrestin-1-Y63-F2Y mutant plasmid or β-arrestin-1-WT was cotransfected with pEVOL-F2YRS plasmids encoding specific *M. jannaschii*tyrosyl amber suppressor tRNA/tyrosyl-tRNA synthtase mutants into BL21 *E. coli* cells. The *E. coli* cells were induced with 0.3mM IPTG for 12 h to allow protein expression in presence of F2Y in the culture medium. A 5 mg sample of β-arrestin-1-Y63-F2Y or

β-arrestin-1-WT protein was purified and subjected to ^{19}F-NMR spectroscopy and mass spectrometry. While the ^{19}F-NMR spectrum of β-arrestin-1-Y63-F2Y showed a single peak at -133.2 ppm (S1a), β-arrestin-1-WT exhibited no detectable ^{19}F-NMR signal (S1b). The molecular weight of β-arrestin-1-Y63-F2Y has a 35.6 Da increase compared with the β-arrestin-1-WT detected by mass spectrometry, in agreement with the adding of 2 fluorine at Y63 positions. These results indicated that the F2Y was specifically inserted into the β-arrestin-1-Y63-F2Y mutant protein.

Supplementary Figure 2.

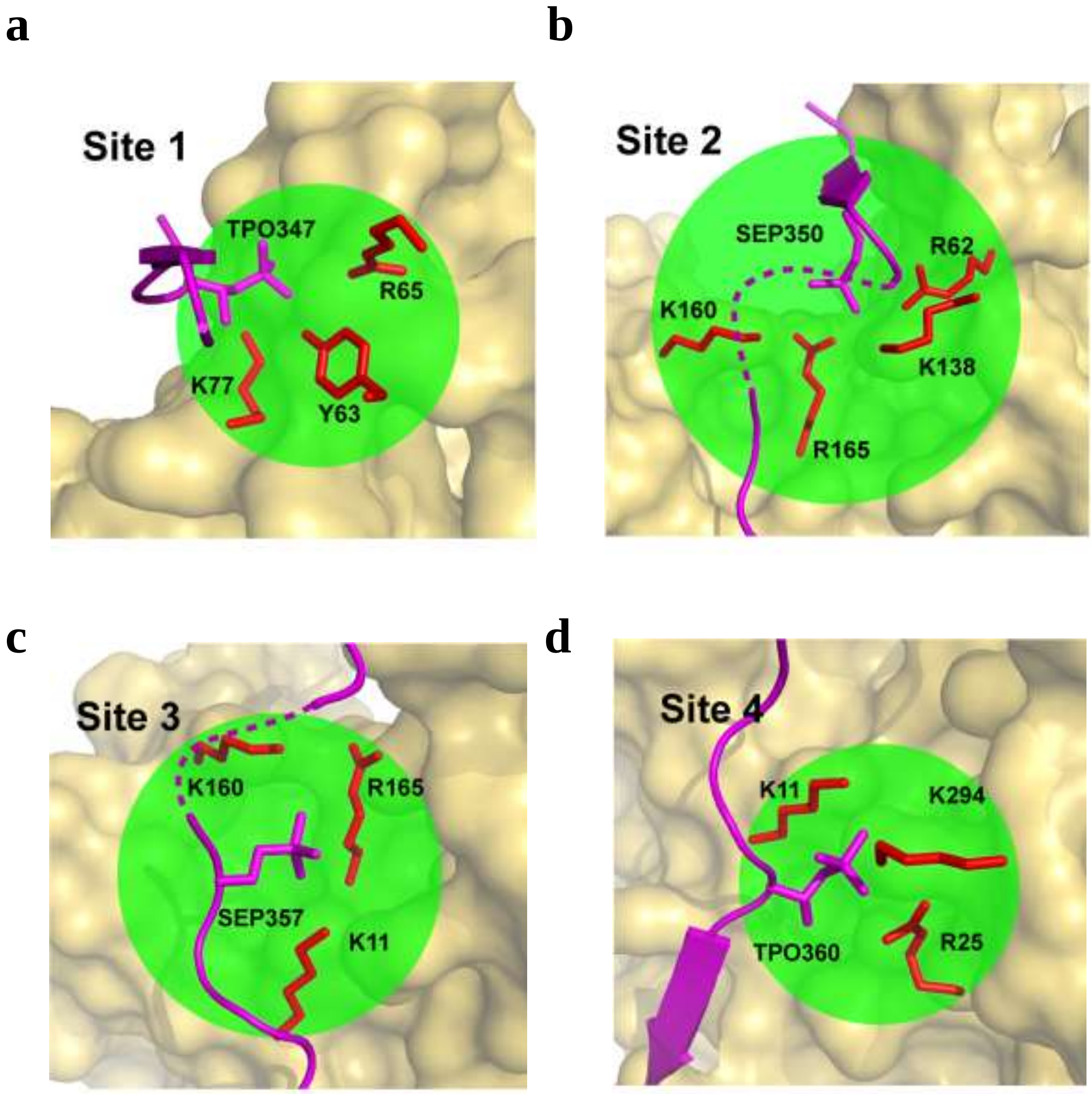

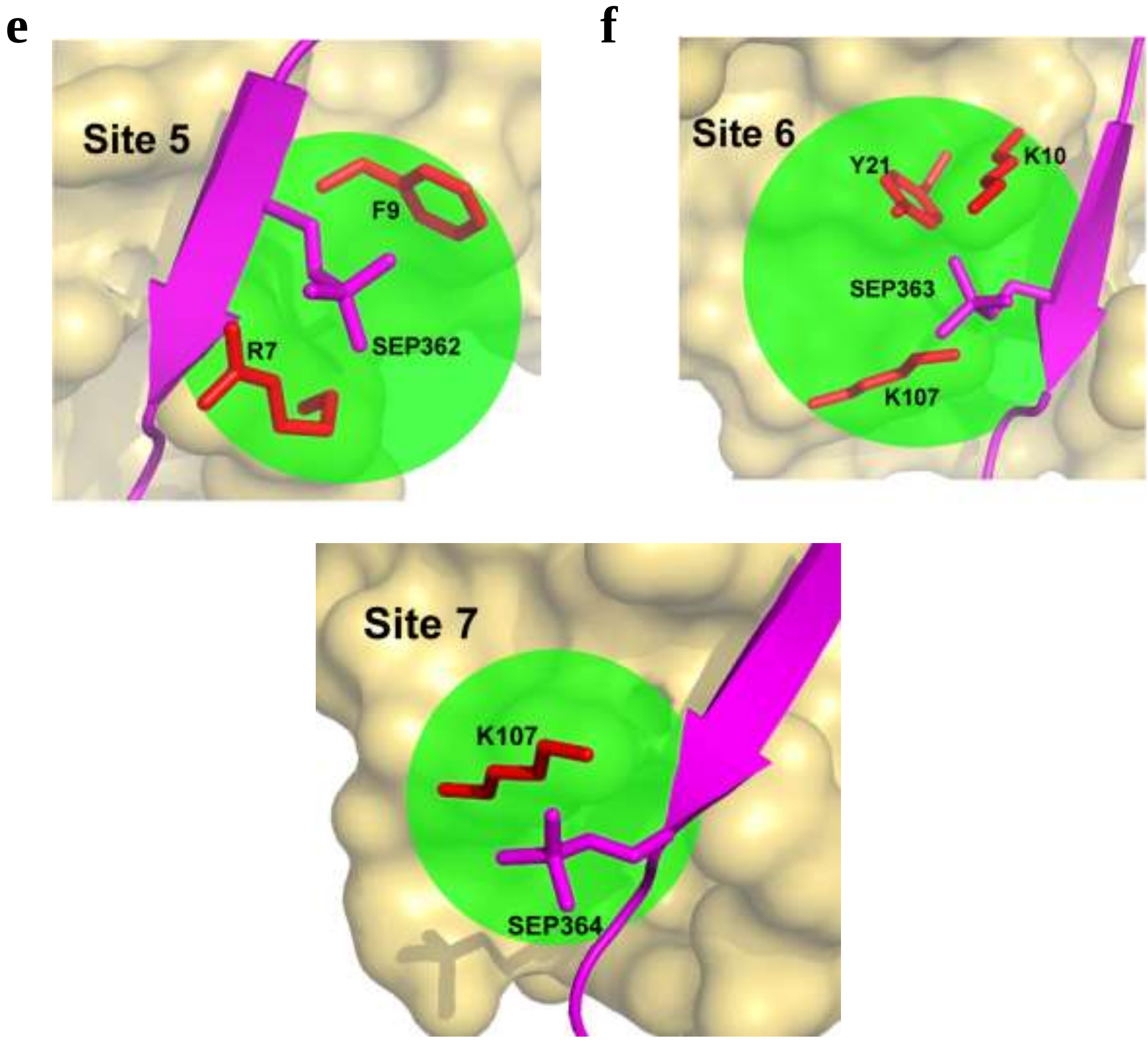

Supplementary Figure 2. Structural representation of the 7 phosphate binding sites in the N-terminal phospho-peptide-binding concave region of β-arrestin-1 (PDB: 4JQI).

Phosphate binding site 1 is surrounded by Y63, R65 and K67(a); phosphate binding site 2 is surrounded by R62, K138, K160 and R165 (b); phosphate binding site 3 is surrounded by K11, K160 and R165 (c); phosphate binding site 4 is surrounded by K11, R25 and K294 (d); phosphate binding site 5 is surrounded by R7 and F9 (e); phosphate binding site 6 is surrounded by K10, Y21 and K107 (f); and phosphate binding site 7 is surrounded by K107 (g); The residues from β-arrestin-1 are shown in red, and V2Rpp is shown in magenta.

Supplementary Figure 3.

a

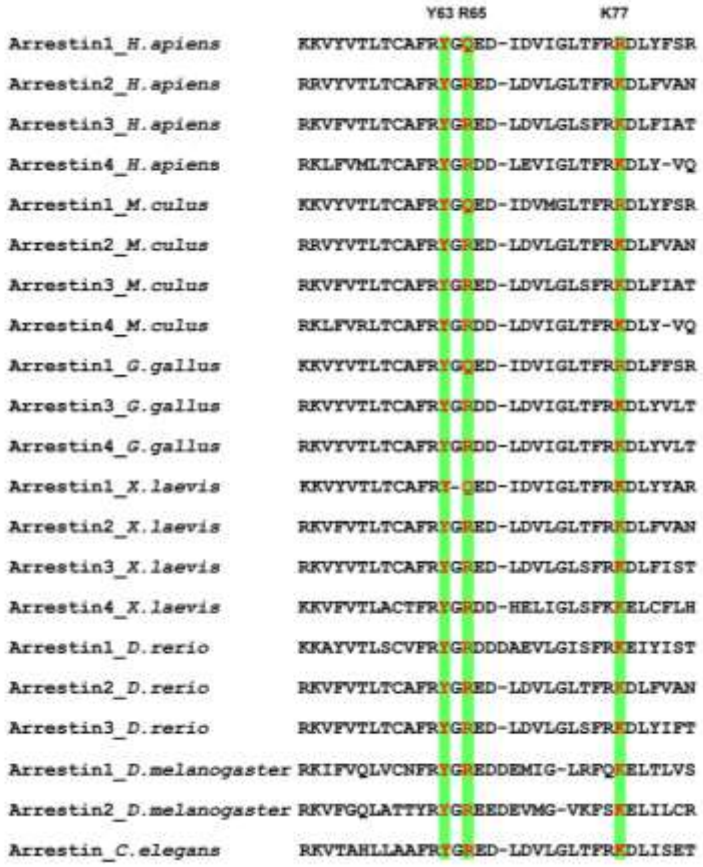

b

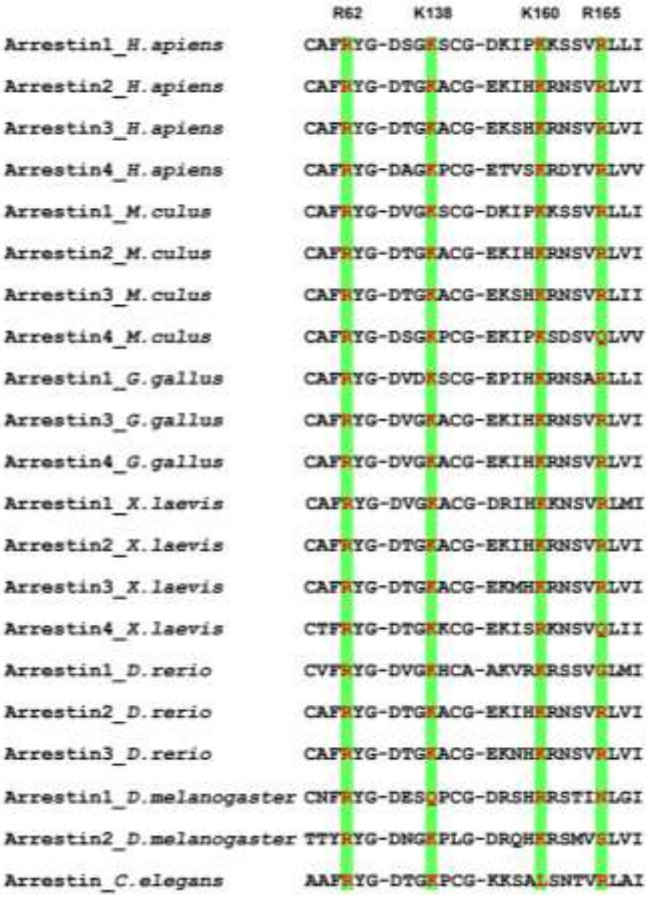

c

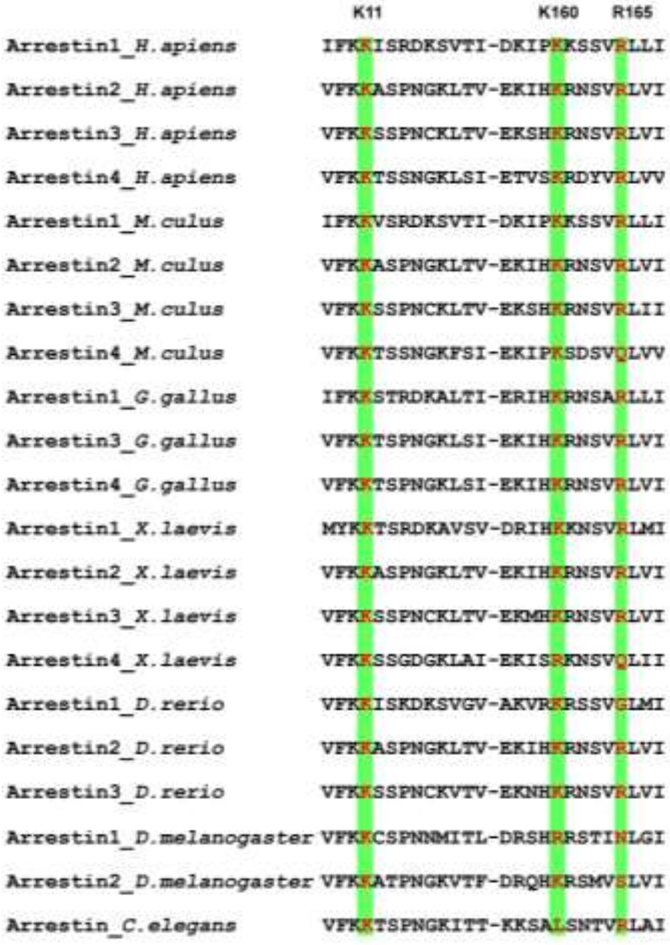
K11
K160
R165
Arrestin1_H.apiens IFKKISRDKSVTI-DKIPKKSSVRLLI
Arrestin2_H.apiens VFKKASPNGKLTV-EKIHKRNSVRLVI
Arrestin3_H.apiens VFKKSSPNCKLTV-EKSHKRNSVRLVI
Arrestin4_H.apiens VFKKTSSNGKLSI-ETVSKRDYVRLVV
Arrestin1_M.culus IFKKVSRDKSVTI-DKIPKKSSVRLLI
Arrestin2_M.culus VFKKASPNGKLTV-EKIHKRNSVRLVI
Arrestin3_M.culus VFKKSSPNCKLTV-EKSHKRNSVRLII
Arrestin4_M.culus VFKKTSSNGKFSI-EKIPKSDSVQLVV
Arrestin1_G.gallus IFKKSTRDKALTI-ERIHKRNSARLLI
Arrestin3_G.gallus VFKKTSPNGKLSI-EKIHKRNSVRLVI
Arrestin4_G.gallus VFKKTSPNGKLSI-EKIHKRNSVRLVI
Arrestin1_X.laevis MYKKTSRDKAVSV-DRIHKKNSVRLMI
Arrestin2_X.laevis VFKKASPNGKLTV-EKIHKRNSVRLVI
Arrestin3_X.laevis VFKKSSPNCKLTV-EKMHKRNSVRLVI
Arrestin4_X.laevis VFKKSSGDGKLAI-EKISRKNSVQLII
Arrestin1_D.rerio VFKKISKDKSVGV-AKVRKRSSVGLMI
Arrestin2_D.rerio VFKKASPNGKLTV-EKIHKRNSVRLVI
Arrestin3_D.rerio VFKKSSPNCKVTV-EKNHKRNSVRLVI
Arrestin1_D.melanogaster VFKKCSPNNMITL-DRSHRRSTINLGI
Arrestin2_D.melanogaster VFKKATPNGKVTF-DRQHKRSMVSLVI
Arrestin_C.elegans VFKKTSPNGKITT-KKSALSNTVRLAI

d

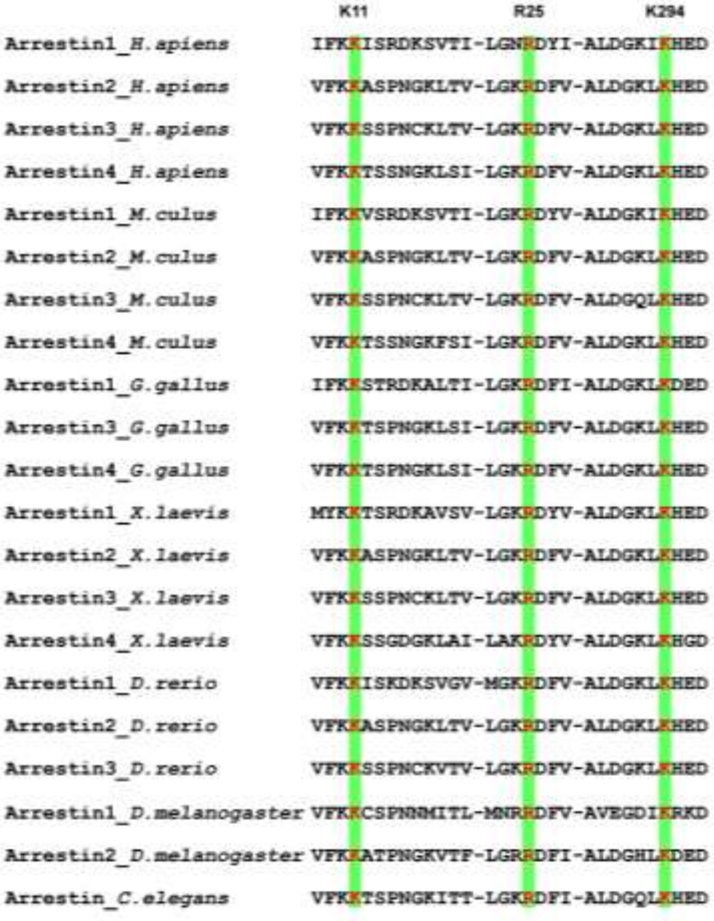
K11
R25
K294
Arrestin1_H.apiens IFKKISRDKSVTI-LGNRDYI-ALDGKIKHED
Arrestin2_H.apiens VFKKASPNGKLTV-LGKRDFV-ALDGKLKHED
Arrestin3_H.apiens VFKKSSPNCKLTV-LGKRDFV-ALDGKLKHED
Arrestin4_H.apiens VFKKTSSNGKLSI-LGKRDFV-ALDGKLKHED
Arrestin1_M.culus IFKKVSRDKSVTI-LGKRDYV-ALDGKIKHED
Arrestin2_M.culus VFKKASPNGKLTV-LGKRDFV-ALDGKLKHED
Arrestin3_M.culus VFKKSSPNCKLTV-LGKRDFV-ALDGQLKHED
Arrestin4_M.culus VFKKTSSNGKFSI-LGKRDFV-ALDGKLKHED
Arrestin1_G.gallus IFKKSTRDKALTI-LGKRDFI-ALDGKLKDED
Arrestin3_G.gallus VFKKTSPNGKLSI-LGKRDFV-ALDGKLKHED
Arrestin4_G.gallus VFKKTSPNGKLSI-LGKRDFV-ALDGKLKHED
Arrestin1_X.laevis MYKKTSRDKAVSV-LGKRDYV-ALDGKLKHED
Arrestin2_X.laevis VFKKASPNGKLTV-LGKRDFV-ALDGKLKHED
Arrestin3_X.laevis VFKKSSPNCKLTV-LGKRDFV-ALDGKLKHED
Arrestin4_X.laevis VFKKSSGDGKLAI-LAKRDYV-ALDGKLKHGD
Arrestin1_D.rerio VFKKISKDKSVGV-MGKRDFV-ALDGKLKHED
Arrestin2_D.rerio VFKKASPNGKLTV-LGKRDFV-ALDGKLKHED
Arrestin3_D.rerio VFKKSSPNCKVTV-LGKRDFV-ALDGKLKHED
Arrestin1_D.melanogaster VFKKCSPNNMITL-MNRRDFV-AVEGDIKRKD
Arrestin2_D.melanogaster VFKKATPNGKVTF-LGRRDFI-ALDGHLKDED
Arrestin_C.elegans VFKKTSPNGKITT-LGKRDFI-ALDGQLKHED

e

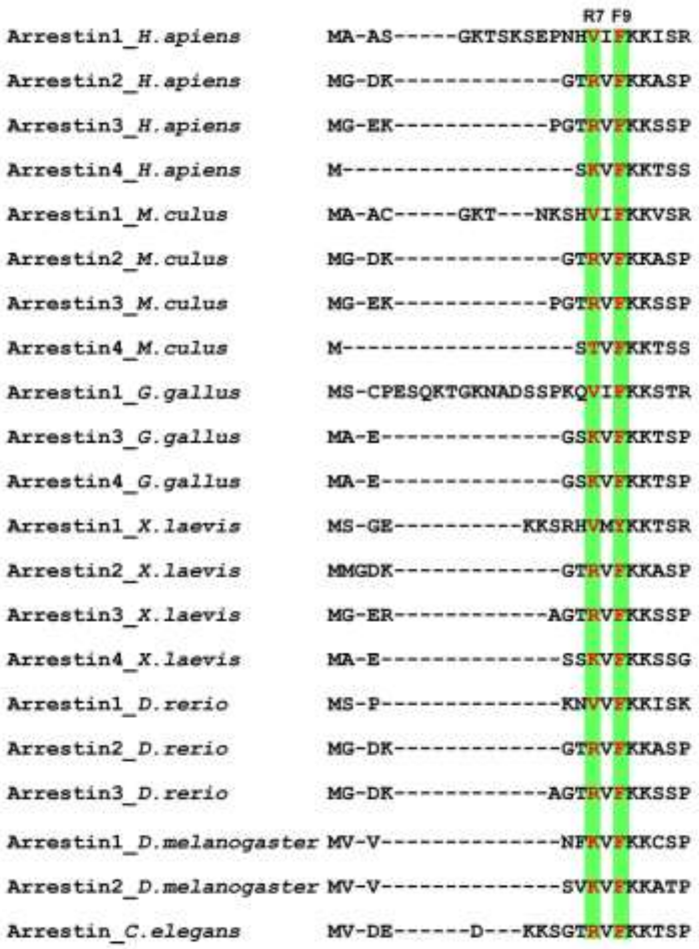
R7 F9
Arrestin1_H.apiens MA-AS-----GKTSKSEPNHVIFKKISR
Arrestin2_H.apiens MG-DK-------------GTRVFKKASP
Arrestin3_H.apiens MG-EK------------PGTRVFKKSSP
Arrestin4_H.apiens M------------------SKVFKKTSS
Arrestin1_M.culus MA-AC-----GKT---NKSHVIFKKVSR
Arrestin2_M.culus MG-DK-------------GTRVFKKASP
Arrestin3_M.culus MG-EK------------PGTRVFKKSSP
Arrestin4_M.culus M------------------STVFKKTSS
Arrestin1_G.gallus MS-CPESQKTGKNADSSPKQVIFKKSTR
Arrestin3_G.gallus MA-E--------------GSKVFKKTSP
Arrestin4_G.gallus MA-E--------------GSKVFKKTSP
Arrestin1_X.laevis MS-GE----------KKSRHVMYKKTSR
Arrestin2_X.laevis MMGDK-------------GTRVFKKASP
Arrestin3_X.laevis MG-ER------------AGTRVFKKSSP
Arrestin4_X.laevis MA-E--------------SSKVFKKSSG
Arrestin1_D.rerio MS-P--------------KNVVFKKISK
Arrestin2_D.rerio MG-DK-------------GTRVFKKASP
Arrestin3_D.rerio MG-DK------------AGTRVFKKSSP
Arrestin1_D.melanogaster MV-V--------------NFKVFKKCSP
Arrestin2_D.melanogaster MV-V--------------SVKVFKKATP
Arrestin_C.elegans MV-DE------D---KKSGTRVFKKTSP

f

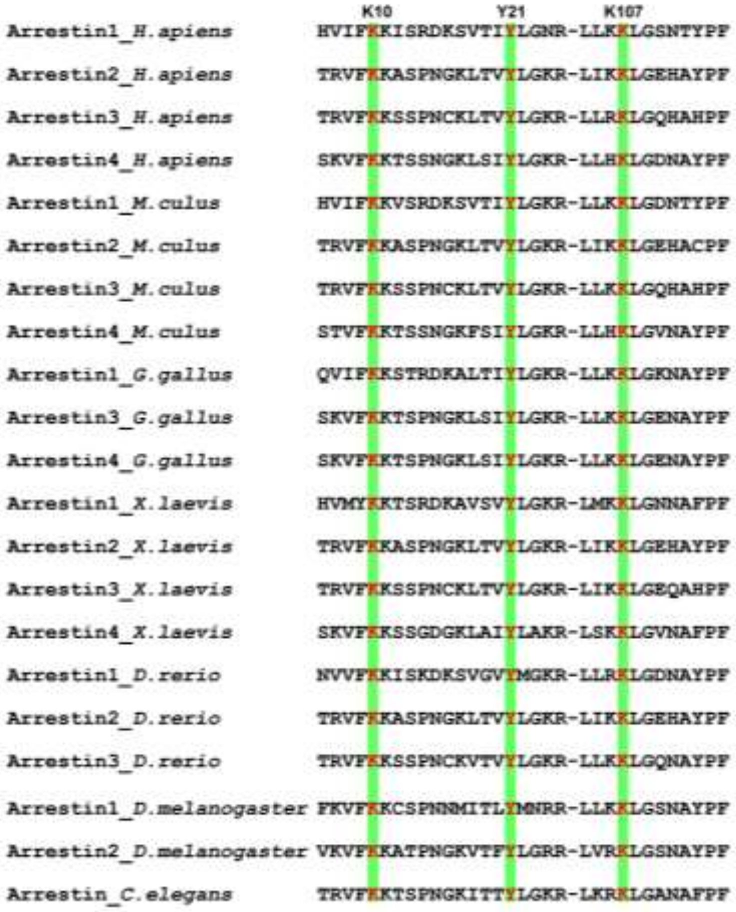
K10 Y21 K107
Arrestin1_H.apiens HVIFKKISRDKSVTIYLGNR-LLKKLGSNTYPF
Arrestin2_H.apiens TRVFKKASPNGKLTVYLGKR-LIKKLGEHAYPF
Arrestin3_H.apiens TRVFKKSSPNCKLTVYLGKR-LLRKLGQHAHPF
Arrestin4_H.apiens SKVFKKTSSNGKLSIYLGKR-LLHKLGDNAYPF
Arrestin1_M.culus HVIFKKVSRDKSVTIYLGKR-LLKKLGDNTYPF
Arrestin2_M.culus TRVFKKASPNGKLTVYLGKR-LIKKLGEHACPF
Arrestin3_M.culus TRVFKKSSPNCKLTVYLGKR-LLKKLGQHAHPF
Arrestin4_M.culus STVFKKTSSNGKFSIYLGKR-LLHKLGVNAYPF
Arrestin1_G.gallus QVIFKKSTRDKALTIYLGKR-LLKKLGKNAYPF
Arrestin3_G.gallus SKVFKKTSPNGKLSIYLGKR-LLKKLGENAYPF
Arrestin4_G.gallus SKVFKKTSPNGKLSIYLGKR-LLKKLGENAYPF
Arrestin1_X.laevis HVMYKKTSRDKAVSVYLGKR-LMKKLGNNAFPF
Arrestin2_X.laevis TRVFKKASPNGKLTVYLGKR-LIKKLGEHAYPF
Arrestin3_X.laevis TRVFKKSSPNCKLTVYLGKR-LIKKLGEQAHPF
Arrestin4_X.laevis SKVFKKSSGDGKLAIYLAKR-LSKKLGVNAFPF
Arrestin1_D.rerio NVVFKKISKDKSVGVYMGKR-LLRKLGDNAYPF
Arrestin2_D.rerio TRVFKKASPNGKLTVYLGKR-LIKKLGEHAYPF
Arrestin3_D.rerio TRVFKKSSPNCKVTVYLGKR-LLKKLGQNAYPF
Arrestin1_D.melanogaster FKVFKKCSPNNMITLYMNRR-LLKKLGSNAYPF
Arrestin2_D.melanogaster VKVFKKATPNGKVTFYLGRR-LVRKLGSNAYPF
Arrestin_C.elegans TRVFKKTSPNGKITTYLGKR-LKRKLGANAFPF

g

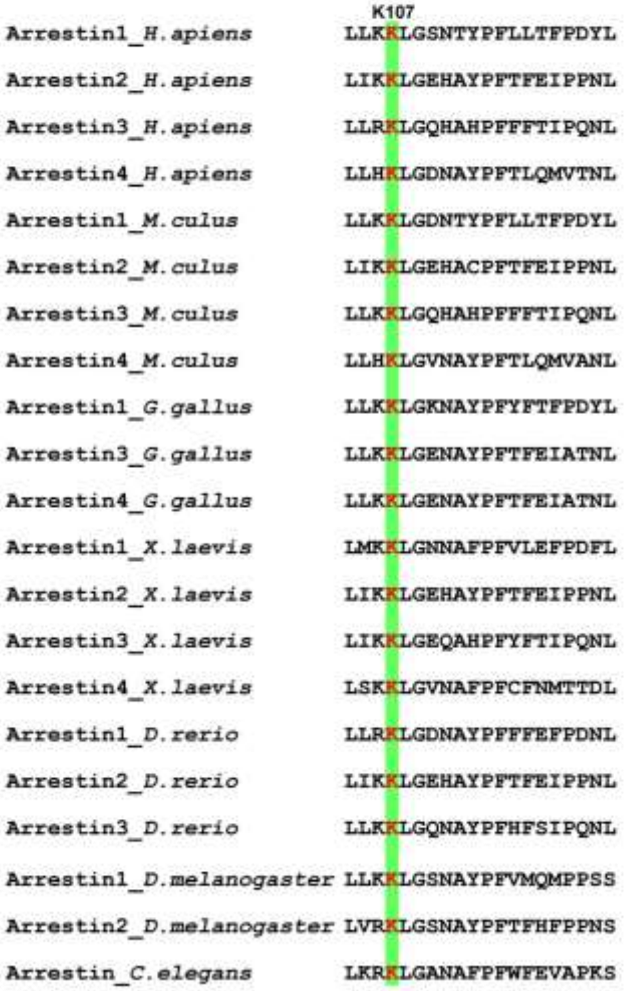

Supplementary Figure 3. Sequence alignment of the residues constituting different phosphate binding site among different arrestin family members across several species.

Panels a to g represent the sequence alignments of the residues constituting phosphate binding sites 1 to 7, respectively. The residues constituting phosphate binding sites 4, 6 and 7 are identical in all arrestin members across different species. Interestingly, these sites are correlated with phospho-peptide-induced clathrin recruitment. The residues constituting binding sites 1, 2, 3, 5 are also conserved in most arrestin members; however, several residues are not identical in different members.

Supplementary Figure 4.

a

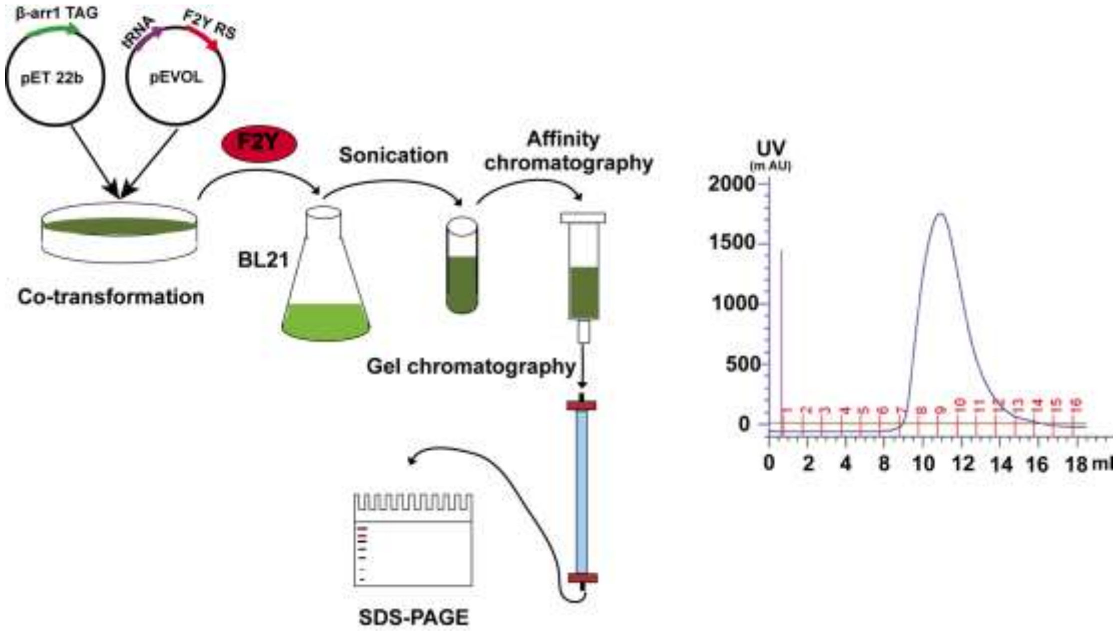

b

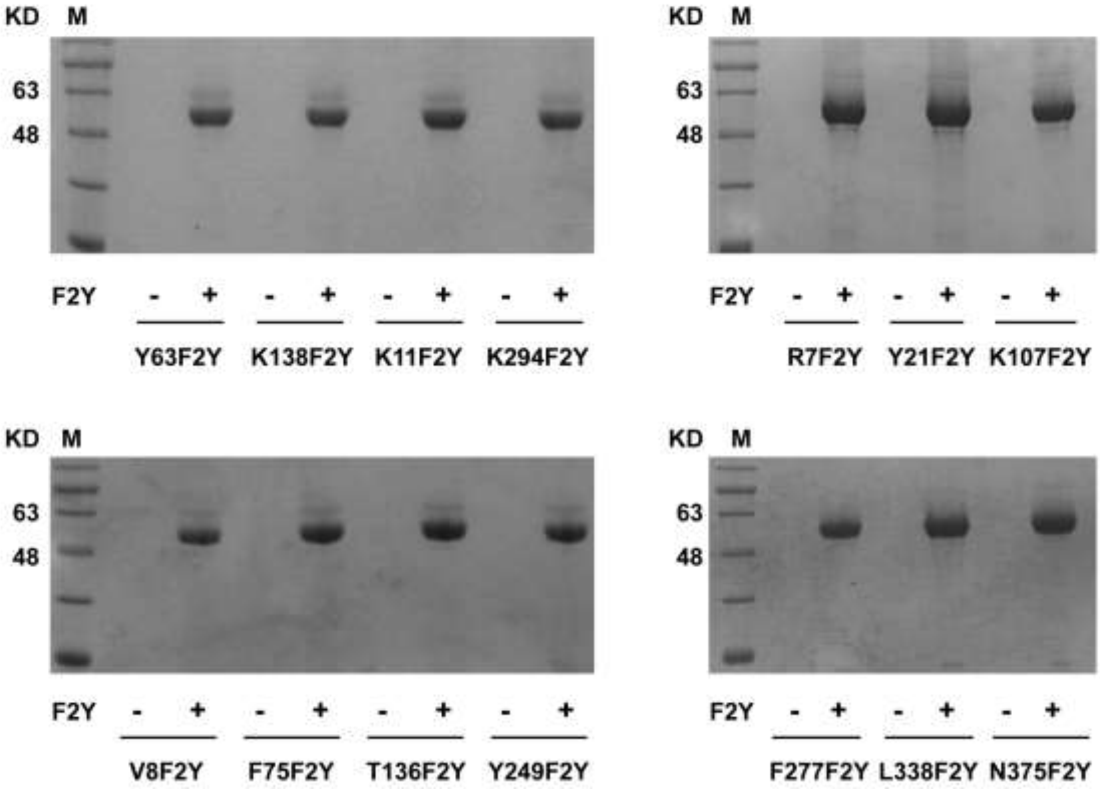

Supplementary Figure 4. Expression and purification of β-arrestin-1 incorporating F2Y.

(a). Left pannel: The procedure for expression and purification of the β-arrestin-1 incorporating theunnatural amino acid F2Y. Right panel: The gel-filtration chromatography of the β-arrestin-1-F2Y protein.

(b). SDS-PAGE analysis of purified β-arrestin-1 mutants incorporating F2Y.

Supplementary Figure 5.

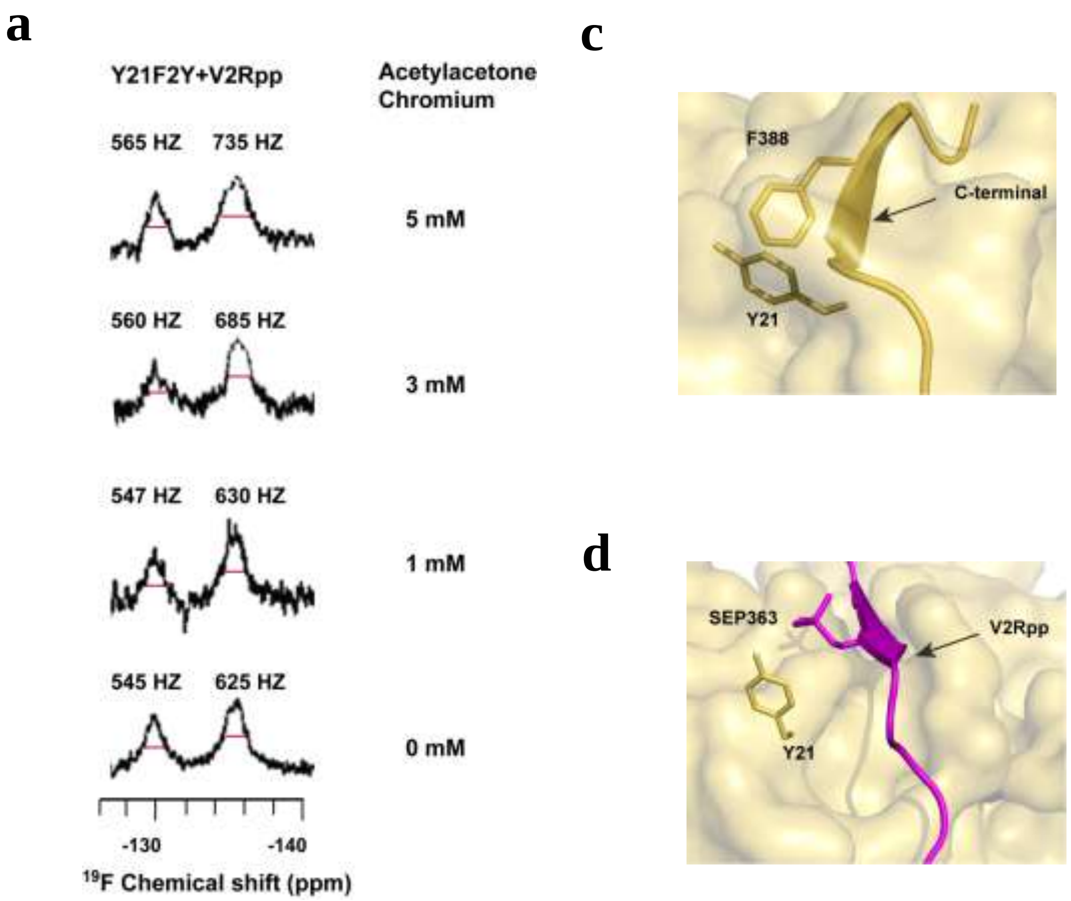

b

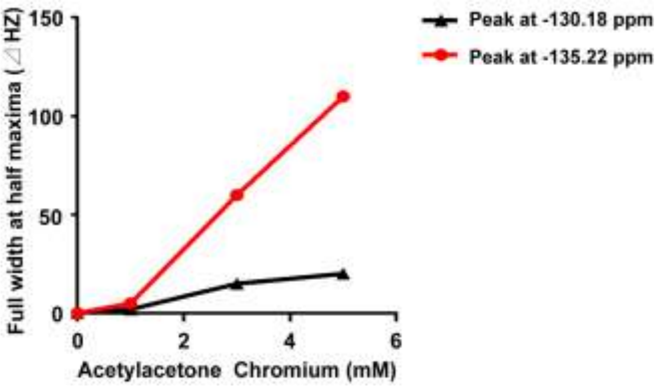

Supplementary Figure 5. Paramagnetic titration experiments revealed two conformational states of the Y21-F2Y in the β-arrestin-1/V2Rpp complex.

(a) The data show the effects of the addition of chromium acetylacetone (Cr) on the 1D-^{19}F-NMR spectra at the F2Y-Y21 position of the β-arrestin-1/V2Rpp complex. The peak at the -130.18 position represents the structural state of F2Y-Y21 in the inactive β-arrestin-1(β-arrestin-1alone), whereas the peak at the -135.22 position represents the structural state of F2Y-Y21 in the active β-arrestin-1(β-arrestin-1/V2Rpp complex).

(b) As the concentration of the Cr increased, the increase of full width at half maximum of the 1D-^{19}F-NMR spectra at the peak of the -130.18 (Δppm=20 HZ) is significantly smaller than the increase at the peak of the -135.22 position (Δppm=110 HZ), indicating a greater protective effect of Cr at the -135.22 position than the -130.18 position.

(c) In the crystal structure of inactive β-arrestin-1 (PDB ID 1G4M), the Y21 was protected from water by F388.

(d) In the crystal structure of β-arrestin-1/V2Rpp complex (PDB ID 4JQI), the F388 of β-arrestin-1 was displaced by the pS363 of the V2Rpp, which was more water accessible. These results disclosed the structural plasticity at the Y21 position of β-arrestin-1 and the replacement of the C tail of β-arrestin-1 after V2Rpp binding.

Supplementary Figure 6.

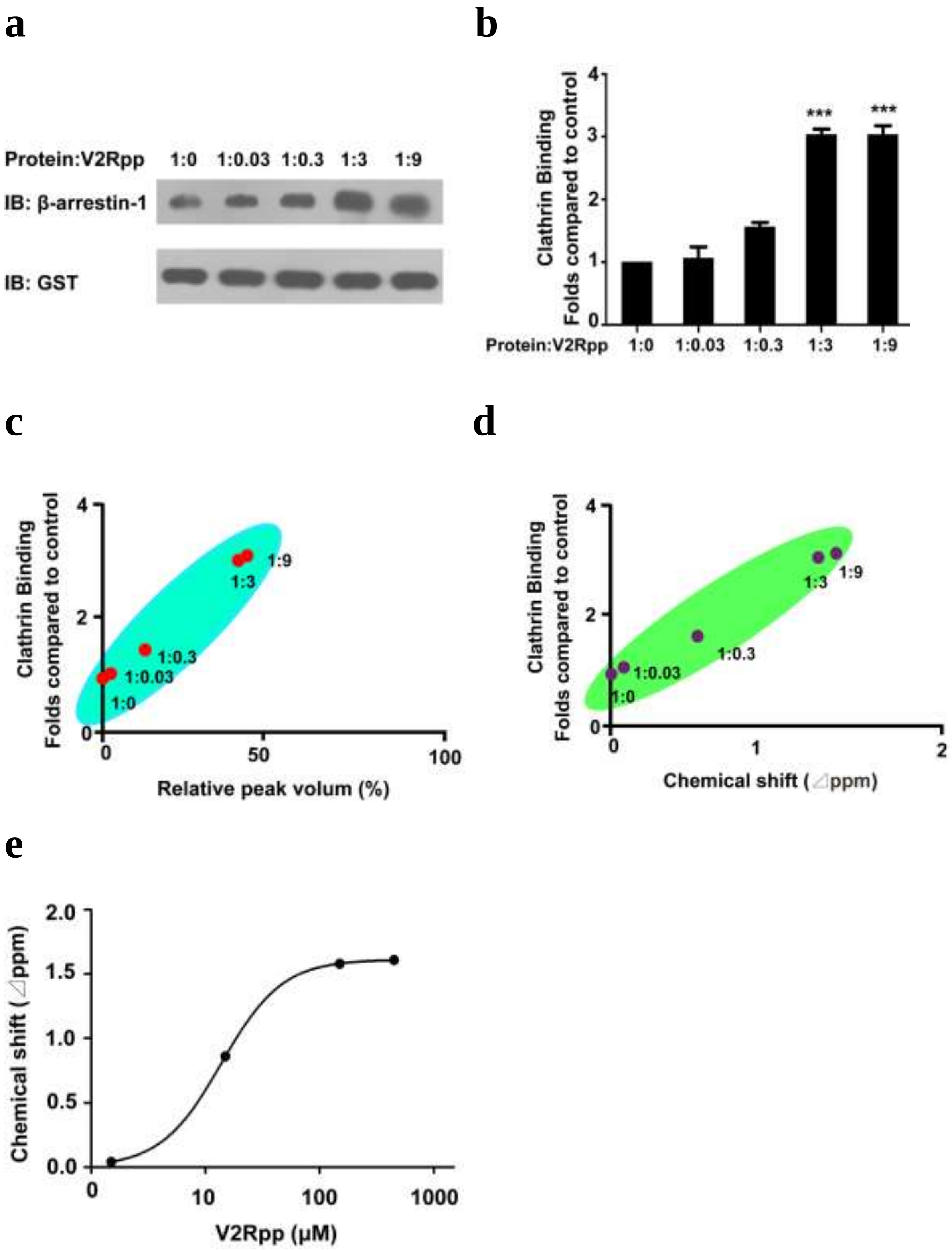

Supplementary Figure 6. The increased peak volume of Y21-F2Y at -135.22 in the ^{19}F-NMR spectrum and the change in amplitude at the Y63-F2Y position are proportional to the increased clathrin binding induced by V2Rpp in a concentration-dependent manner.

(a) Representative western blot showing the effects of V2Rpp with different concentrations.

(b) Quantitative analysis of the amount of clathrin associated with β-arrestin-1 from Figure S7A, from at least 3 independent experiments. ***, $p<0.01$, V2Rpp stimulation compared with non-phospho-peptide stimulation.

(c) The *x*-axis represents the relative peak volume of Y21-F2Y at -135.22 in relation to the sum of the total peak volume of Y21-F2Y （-130.18 and -135.22） in the ^{19}F-NMR spectrum. The *y*-axis depicts V2Rpp-induced clathrin binding compared with the control.

(d) The *x*-axis represents the change in the amplitude of the chemical shift at the Y63-F2Y position in the ^{19}F-NMR spectrum. The *y*-axis depicts V2Rpp-induced clathrin binding compared with the control.

(e) The *x*-axis represents the concentration of V2Rpp and the *y*-axis depictsthe change in the amplitude of the chemical shift at the Y63-F2Y position in the ^{19}F-NMR spectrum.

Supplementary Figure 7.

a

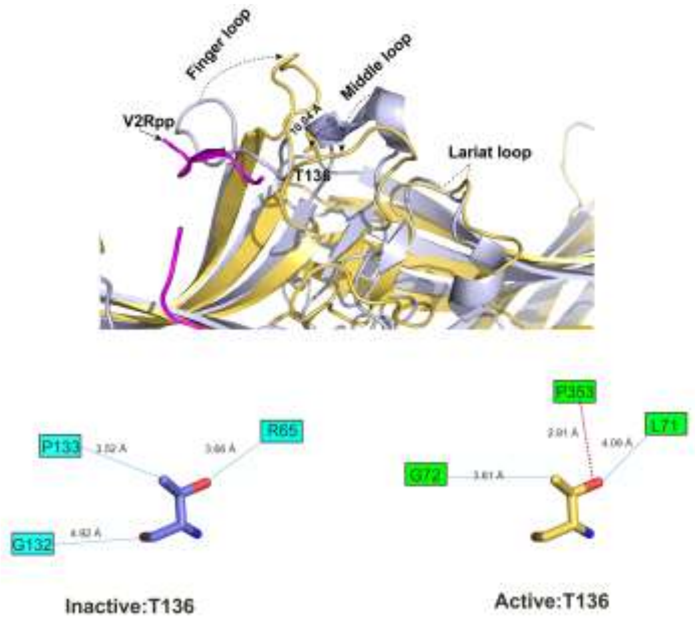

b

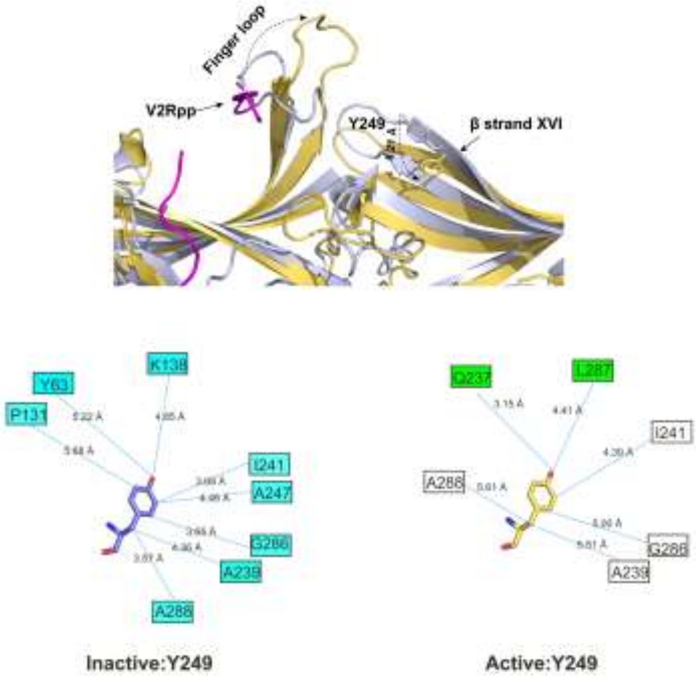

c

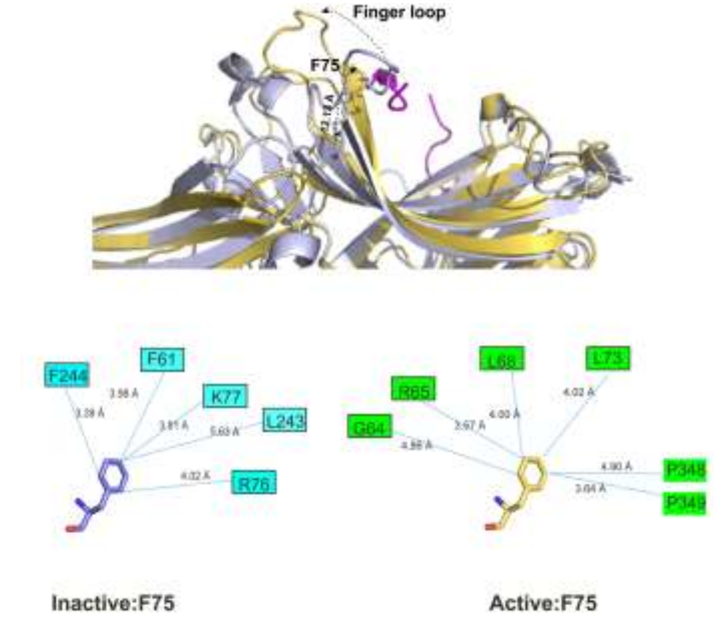
Finger loop
F75
F244
F61
K77
L243
R76
L68
L73
R65
G64
P348
P349
Inactive:F75
Active:F75

d

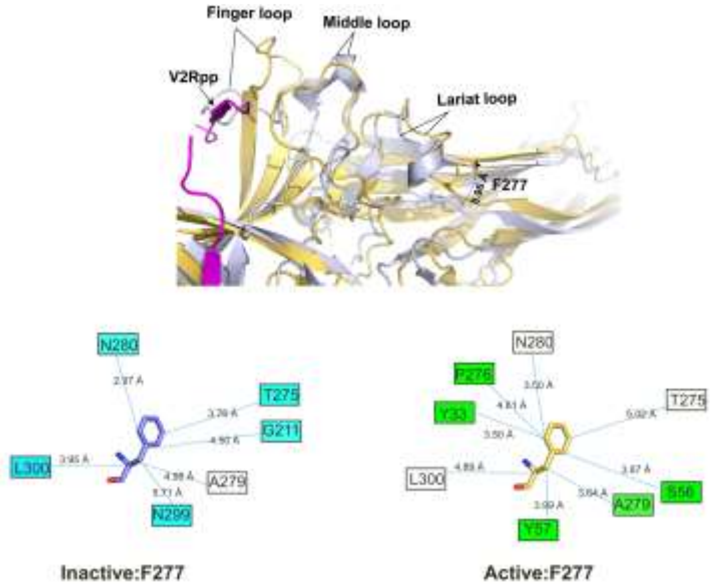
Finger loop
Middle loop
V2Rpp
Lariat loop
F277
N280
T275
G211
L300
A279
N299
P276
Y33
S56
Y57
Inactive:F277
Active:F277

e

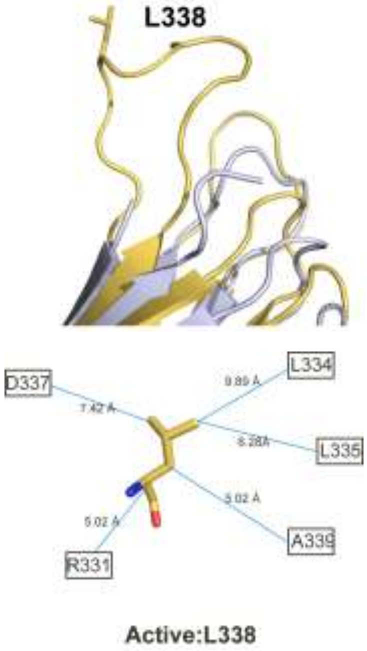
L338
D337
L334
L335
A339
R331
Active:L338

f

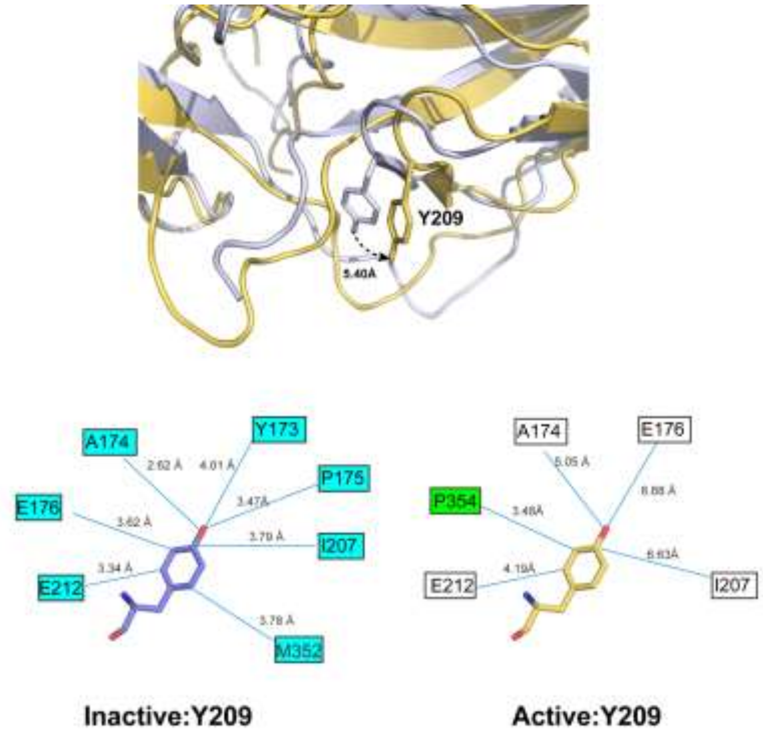
Y209
5.40Å
A174
Y173
P175
E176
I207
E212
M352
Inactive:Y209
A174
E176
P354
E212
I207
Active:Y209

Supplementary Figure 7. Comparison of the localized F2Y incorporation positions in the crystal structures of inactive β-arrestin-1 (PDB: 1G4M) and V2R-phospho-peptide-activated β-arrestin-1 (PDB: 4JQI) other than the phosphate binding sites.

Upper panel: Each upper panel shows the structural comparison of inactive β-arrestin-1 and V2R-phospho-peptide activated β-arrestin-1 in cartoon mode. Inactive β-arrestin-1 structure is depicted in gray, the phospho-peptide is in magenta and the β-arrestin-1occupied by the phospho-peptide is in yellow.

Lower panels: The lower panels are the schematic representations of interactions between specific F2Y incorporation position with their surrounding residues (left, inactive; right, active). Residues with increased distance are coloured in cyan and residues with decreased distance or forming new interactions are coloured in green. The phospho-residue from V2R-phospho-peptide (V2Rpp) is coloured in red.

(a). F75: Binding of V2Rpp with β-arrestin-1 induced significant conformational change at the F75. The F75 moved away from the F61, R76, K77, L243 and F244 and formed new interactions with the G64, R65, L68, L73, P348 and P349.

(b). T136: Binding of V2Rpp with β-arrestin-1 moved the T136 away from the R65, G132, P133 and get the T136 close to the L71,G72 and P353.

(c). Y249：Interaction of V2Rpp with β-arrestin-1 induced the Y249 to move away from Y63, P131, K138, A239, I241, A247, G286 and A288 and form new interactions with the Q237 and L287.

(d). F277: Binding of V2Rpp with β-arrestin-1 caused the F277 to move away from the G211, T275, N280, N299 and L300, get close to the A279 and form new interactions with the Y33, S56, Y57 and P276.

(e) L338: The electron density of L338 is not defined in the crystal structure of inactive β-arrestin-1. In V2Rpp-bound β-arrestin-1, L338 forms hydrophobic interactions with L335 and A339 and is close to R331, L334 and D337.

(f) Y209: Binding of V2Rpp with β-arrestin-1 caused the F277 to move away from the Y173, A174, P175, E176, I207, E212 and M352 and form new interactions with the P354.

Supplementary Figure 8.

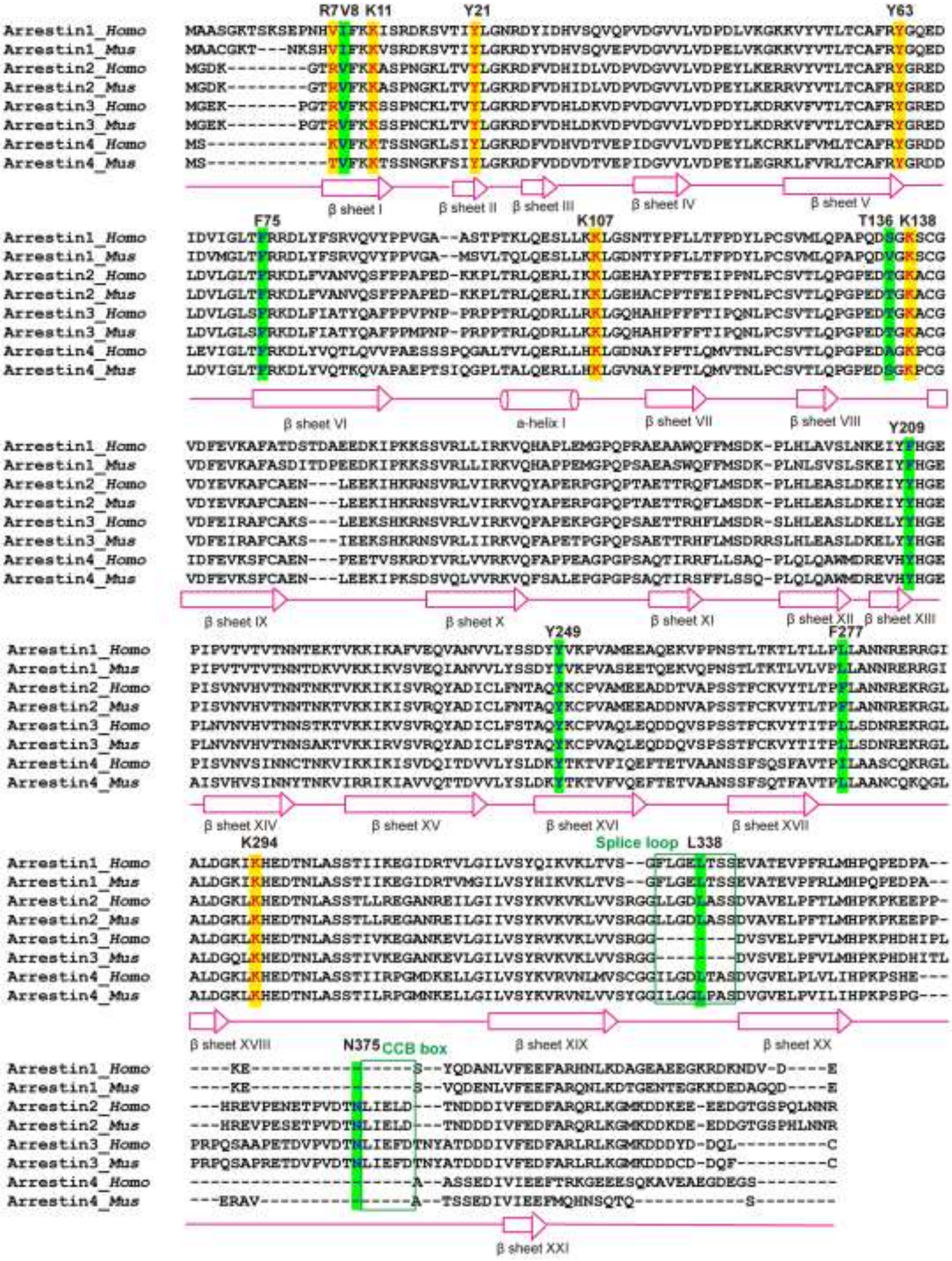

Supplementary Figure 8. Sequence alignment of arrestin family members from human and mice.

F2Y-incorporated positions at phosphate binding sites are coloured in red and highlighted in yellow. The F2Y-incorporated positions that are not located at phosphate binding sites are highlighted in green. Notably, while the long isoform of β-arrestin-1 contains both regions, the cone and rod arrestins lack the CCB box, and

β-arrestin-2 and the short form of β-arrestin-1 lack the splice loop, suggesting potentially different modes of arrestin-subtype/clathrin interactions.

Supplementary Figure 9.

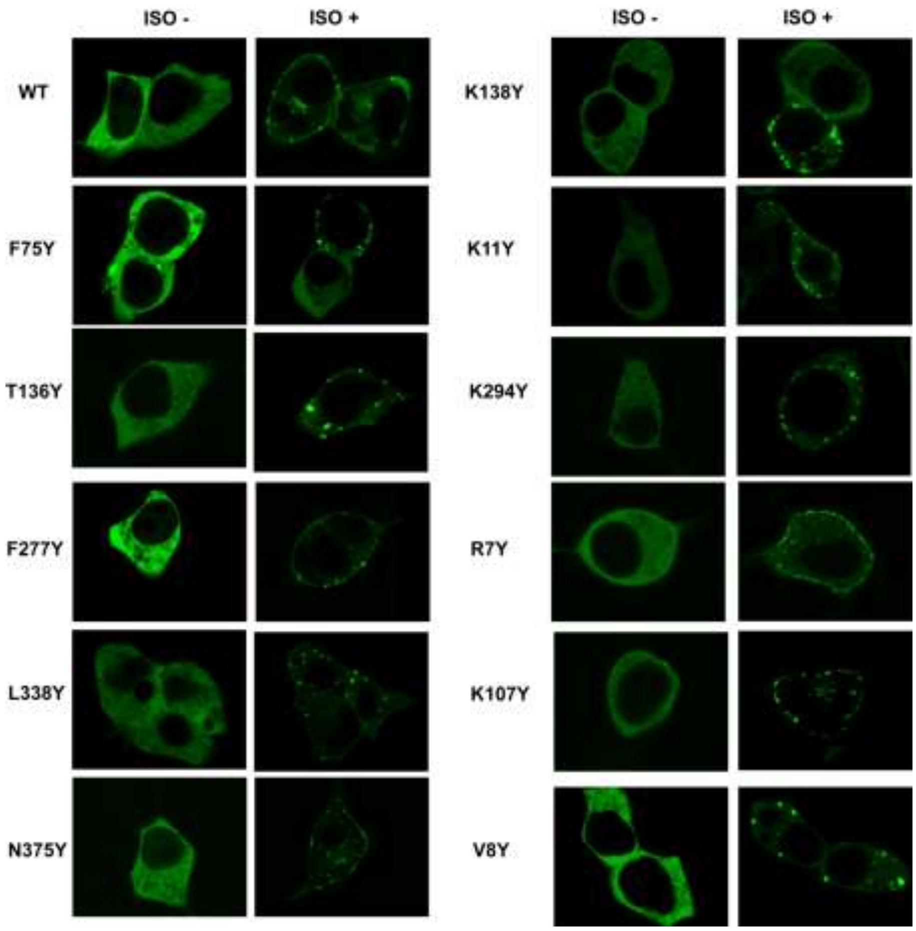

Supplementary Figure 9. Effects of β-arrestin-1 mutants on isoproterenol (ISO)-induced β-arrestin-1 translocation.

The plasmids encoding specific β-arrestin-1 mutants with C-terminal YFP tag were transfected into β2-AR stable cell lines. Similar to wild type β-arrestin-1, the mutated versions F75Y, T136Y, F277Y, L338Y, N375Y, K138Y, K11Y, K294Y, R7Y, K107Y translocated to the perimembrane region after ISO stimulation. It is worth noting that the T136Y, N375Y, L338Y and F277Y mutants may not fully correspond to the results of the functional analysis of F2Y-T136, F2Y-N375, F2Y-L338 and F2Y-F277 due to the difference in pKa between Y and F2Y.

Supplementary Figure 10.

a

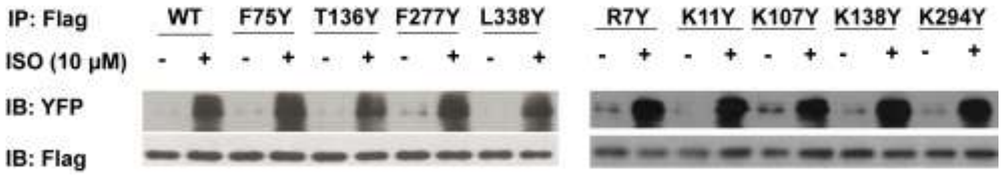

b

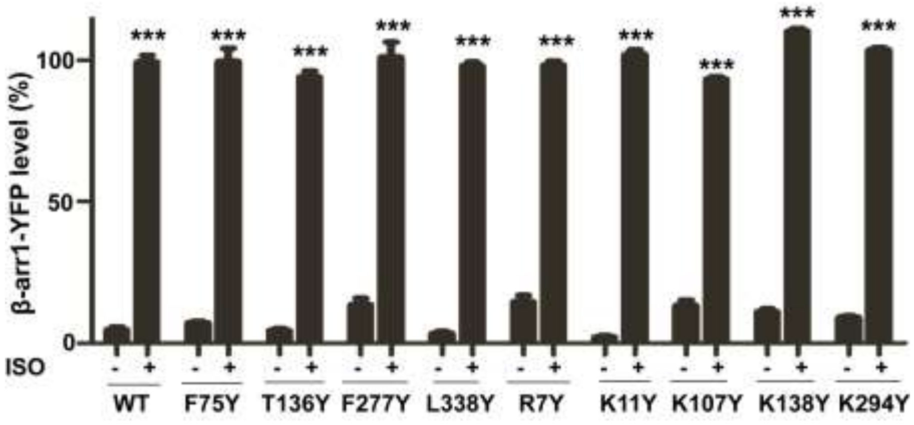

Supplementary Figure 10. Effects of β-arrestin-1 mutations on ISO-induced β2AR/β-arrestin-1 complex formation.

(a).HEK293 cells were co-transfected with Flag-β2AR and β-arrestin-1-YFP mutants, as indicated in the figures. The cells were starved before stimulation with ISO (10 μM) for 10 min. The β2AR/β-arrestin-1 complex was immunoprecipitated using anti-Flag affinity agarose, and formation of the complex was monitored using western blotting and specific anti-YFP antibodies. It is worth noting that the T136Y, L338Y and F277Y mutants may not fully correspond to the results of the functional analysis of F2Y-T136, F2Y-L338 and F2Y-F277 due to the difference in pKa between Y and F2Y.

(b).The western blot signals of β-arrestin-1 bound to β2AR (A) were quantified and are shown as columns. ***, $p<0.005$; ISO treated cells are compared with unstimulated cells. All of the tested β-arrestin-1 mutants bound to β2AR after ISO stimulation.

Supplementary Figure 11.

a

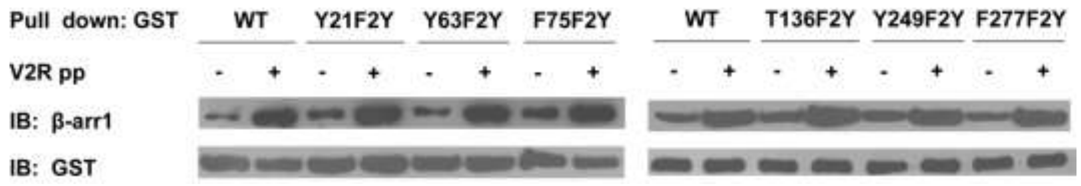

b

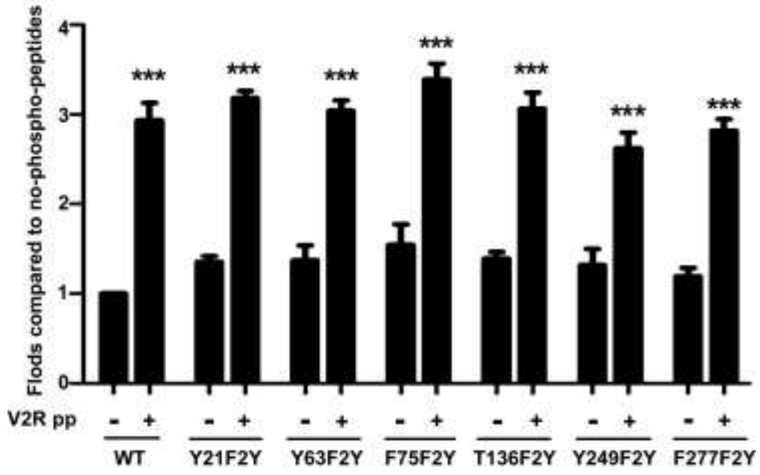

c

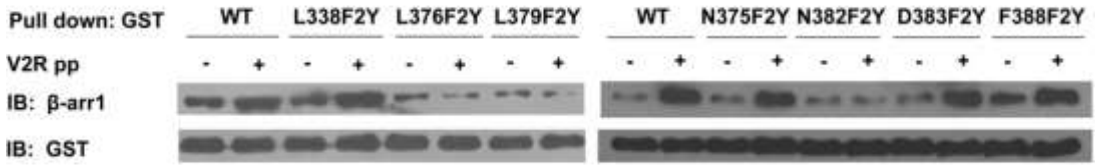

d

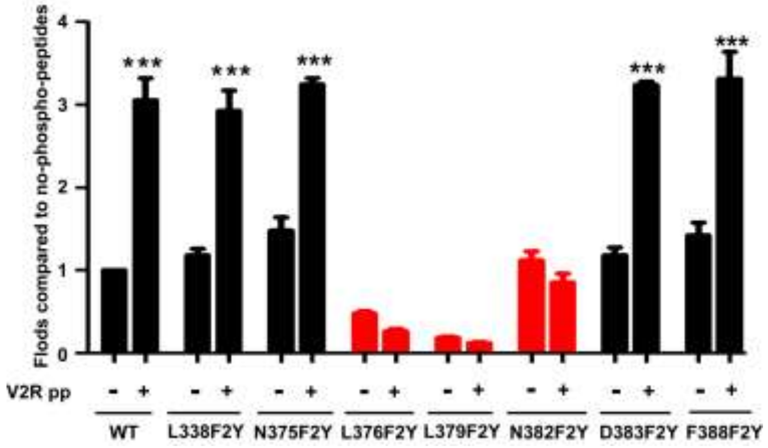

e

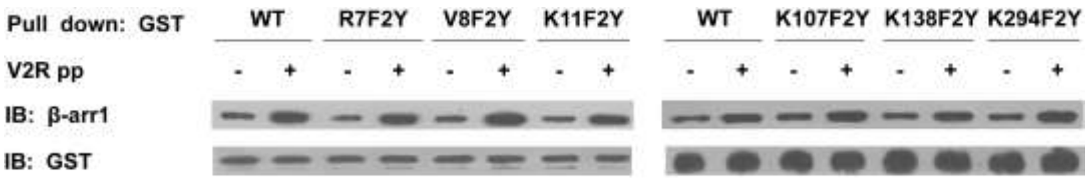

f

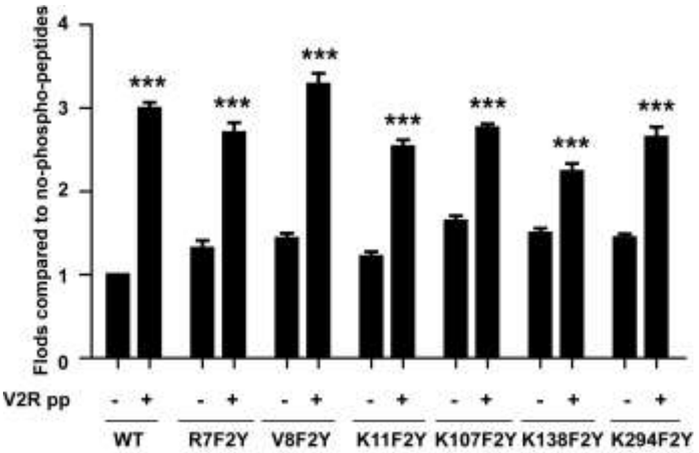

Supplementary Figure 11. Effects of F2Y mutants of β-arrestin-1 on V2Rpp promoted β-arrestin-1/clathrin complex formation.

(a/c/e) β-arrestin-1-F2Y mutants (300 nM) were incubated with equal concentrations of GST-clathrin and V2R-phospho-peptides. The complexes were pulled down using GST beads, and the amount of β-arrestin-1 bound to clathrin was determined using a specific β-arrestin-1 antibody.

(b, d or f) Western blot signals of β-arrestin-1 bound to clathrin (a, c or e) were quantified. ***, $p<0.005$; V2Rpp stimulated β-arrestin-1/clathrin complex formation are compared with basal β-arrestin-1/clathrin complex formation. Most F2Y β-arrestin-1 mutants promoted β-arrestin-1/clathrin complex formation in the presence of V2R-phospho-peptides, whereas three β-arrestin-1 mutations (L376-F2Y, L379-F2Y and N382-F2Y) in the classic clathrin binding (CCB) box did not exhibit this ability. Therefore, the L376-F2Y, L379-F2Y and N382-F2Y were not used as ^{19}F-NMR probes for further studies.

Supplementary Figure 12.

a

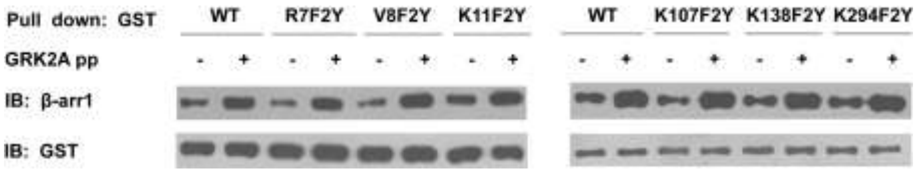

b

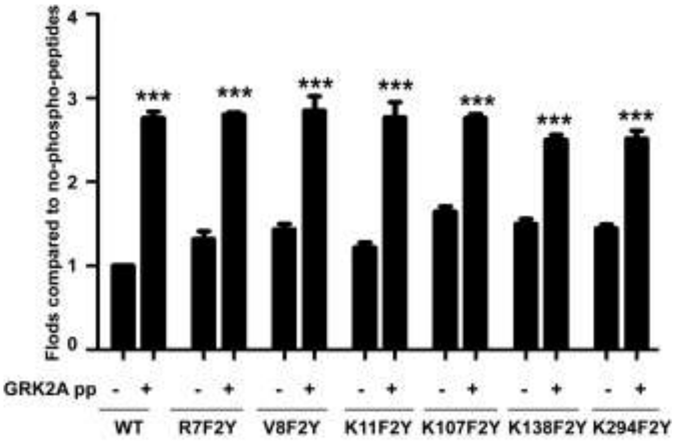

c

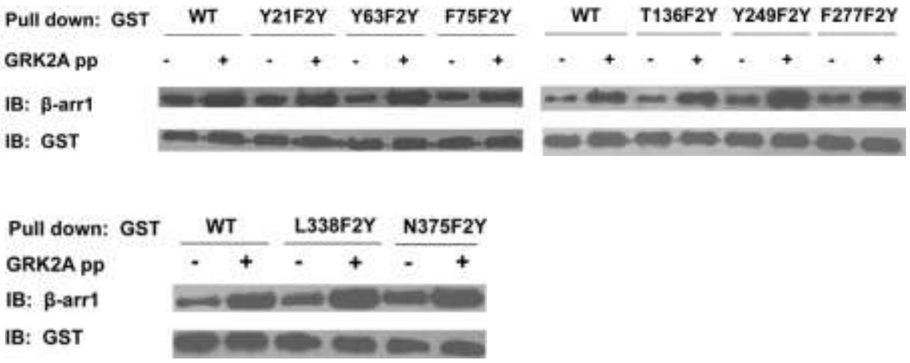

d

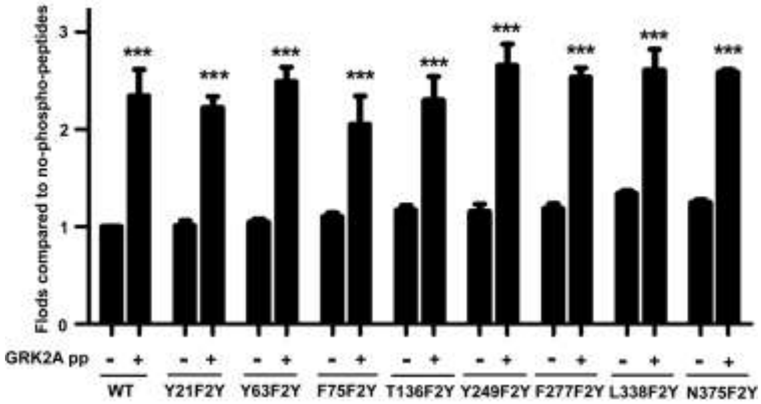

e

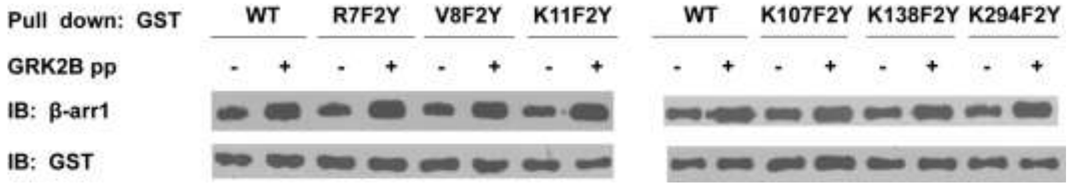

Pull down: GST
WT
R7F2Y
V8F2Y
K11F2Y
WT
K107F2Y
K138F2Y
K294F2Y
GRK2B pp
IB: β-arr1
IB: GST

f

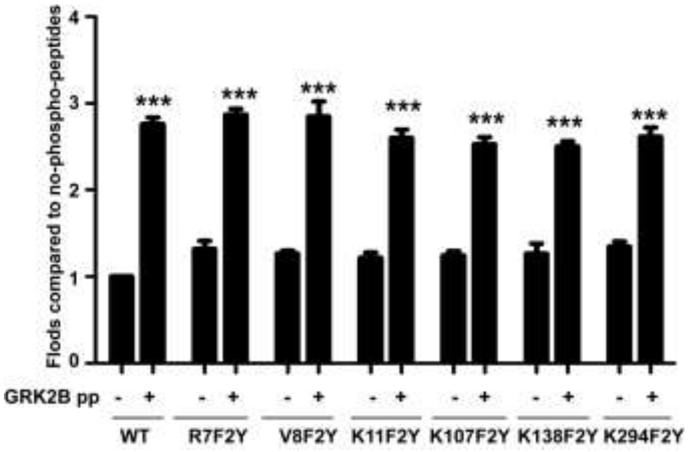

Flods compared to no-phospho-peptides
GRK2B pp
WT
R7F2Y
V8F2Y
K11F2Y
K107F2Y
K138F2Y
K294F2Y

g

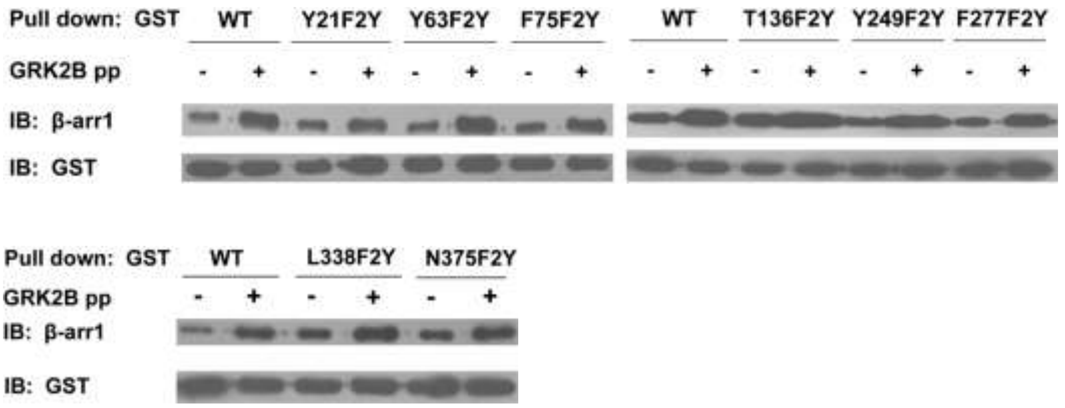

Pull down: GST
WT
Y21F2Y
Y63F2Y
F75F2Y
WT
T136F2Y
Y249F2Y
F277F2Y
GRK2B pp
IB: β-arr1
IB: GST
Pull down: GST
WT
L338F2Y
N375F2Y
GRK2B pp
IB: β-arr1
IB: GST

h

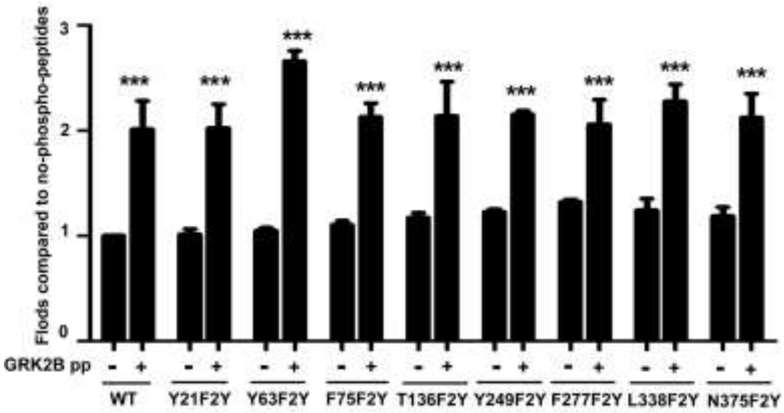

Flods compared to no-phospho-peptides
GRK2B pp
WT
Y21F2Y
Y63F2Y
F75F2Y
T136F2Y
Y249F2Y
F277F2Y
L338F2Y
N375F2Y

Supplementary Figure 12. Effects of F2Y mutants of β-arrestin-1 on GRK2 phospho-peptide-promoted β-arrestin-1/clathrin complex formation.

(a/c/e/g) β-arrestin-1-F2Y mutants (10 μM) were incubated with equal concentrations of GST-clathrin and GRK2A-phospho-peptide (a/c) or GRK2B-phospho-peptide (e/g). The complexes were pulled down using GST beads, and the amount of β-arrestin-1 bound to clathrin was determined using a specific β-arrestin-1 antibody.

(b, d, f, h) Western blot signals of β-arrestin-1 bound to clathrin (a, c, e or g) were quantified. ***, $p<0.005$; GRK2-phospho peptide stimulated β-arrestin-1/clathrin complex formation are compared with basal β-arrestin-1/clathrin complex formation.

Supplementary Figure 13.

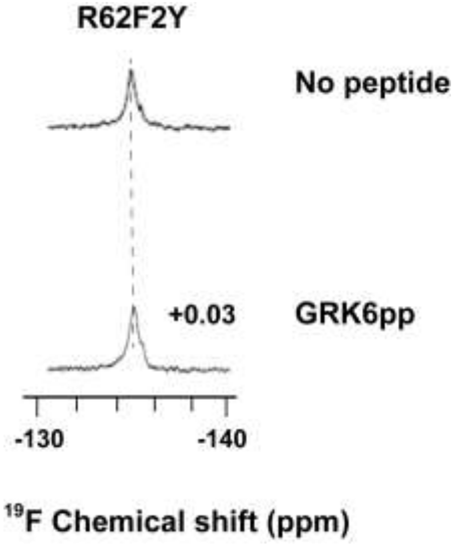

Supplementary Figure 13. Effects of binding of GRK6pp on the ^{19}F-NMR spectrum on β-arrestin-1-R62-F2Y phospho-sensing probe. No significant chemical shift was detected.

Supplementary Figure 14.

a

Peptide	Sequence
GRK6	^{343}RRSIKAYGNGY pS pS NGNTG E QSGYHV E Q ^{370}
GRK6-pS355S	^{343}RRSIKAYGNGY S pS NGNTG E QSGYHV E Q ^{370}
GRK6-pS356S	^{343}RRSIKAYGNGY pS S NGNTG E QSGYHV E Q ^{370}
GRK6-E369A	^{343}RRSIKAYGNGY pS pS NGNTG E QSGYHV A Q ^{370}

b

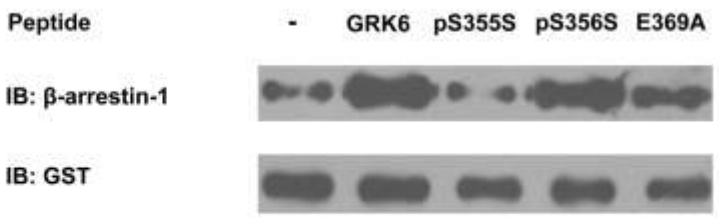

c

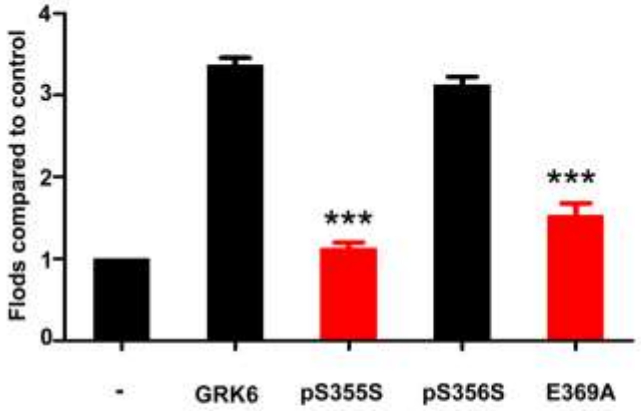

Supplementary Figure 14. Effects of GRK6pp mutants on phospho-peptide-promoted β-arrestin-1/SRC complex formation.

(a). Sequence information of the specific GRK6pp mutants. Phospho-Ser or Glu is mutated to Ser or Ala respectively.

(b) 300nM GRK6pp or specific GRK6pp mutants were incubated with equal concentrations of β-arrestin-1 WT and GST-SRC-3D. The complexes were pulled down using GST beads, and the amount of β-arrestin-1 bound to SRC was determined using a specific β-arrestin-1 antibody. Representative western blot from at least three experiments is shown.

(c) Western blot signals of β-arrestin-1 bound to SRC (b) were quantified. ***, $p<0.005$; β-arrestin-1/SRC complex formation with or without different phospho-peptides are compared with basal β-arrestin-1/SRC complex formation.

Supplementary Figure 15.

a

Peptide	Sequence
GRK2B	357 NGN pT G E Q pS GYHV E Q E K E NKLLC ED LPGTE 385
GRK2B-pT360T	357 NGN T G E Q pS GYHV E Q E K E NKLLC ED LPGTE 385
GRK2B-E362A	357 NGN pT G A Q pS GYHV E Q E K E NKLLC ED LPGTE 385
GRK2B-pS364S	357 NGN pT G E Q S GYHV E Q E K E NKLLC ED LPGTE 385
GRK2B-E369A	357 NGN pT G E Q pS GYHV A Q E K E NKLLC ED LPGTE 385
GRK2B-E371A	357 NGN pT G E Q pS GYHV E Q A K E NKLLC ED LPGTE 385
GRK2B-E373A	357 NGN pT G E Q pS GYHV E Q E K A NKLLC ED LPGTE 385
GRK2B-E379/D380A	357 NGN pT G E Q pS GYHV E Q E K E NKLLC AA LPGTE 385

b

c

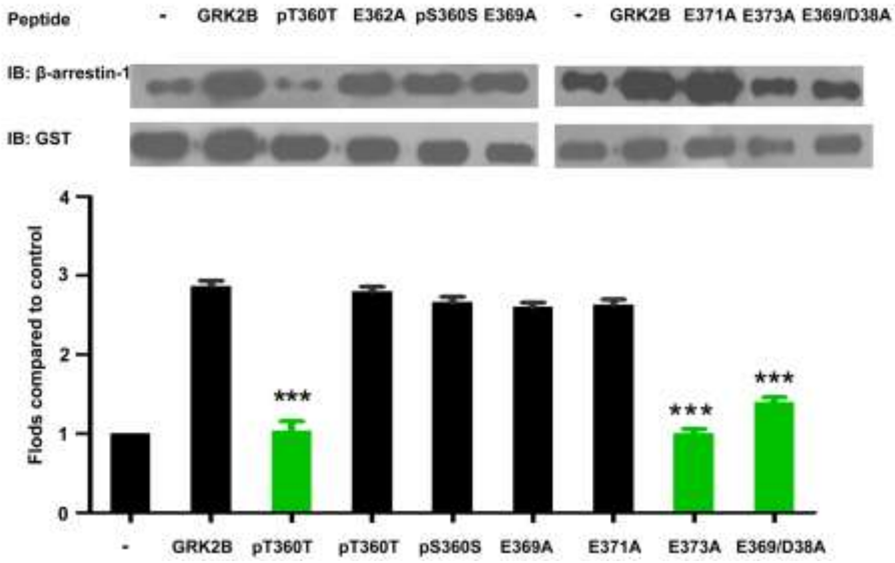

Supplementary Figure 15. Effects of GRK2Bpp mutants on phospho-peptide-promoted β-arrestin-1/clathrin complex formation.

(a). Sequence information of the specific GRK2Bpp mutants. Phospho-Ser or Asp/Glu is mutated to Ser or Ala respectively.

(b) 300nM GRK2Bpp or specific GRK2Bpp mutants were incubated with equal concentrations of β-arrestin-1 WT and GST-clathrin. The complexes were pulled down using GST beads, and the amount of β-arrestin-1 bound to clathrin was

determined using a specific β-arrestin-1 antibody. Representative western blot from at least three experiments is shown.

(c) Western blot signals of β-arrestin-1 bound to clathrin (b) were quantified. ***, $p<0.005$; β-arrestin-1/clathrin complex formation with or without different phospho-peptides are compared with basal β-arrestin-1/clathrin complex formation.

Supplementary Figure 16.

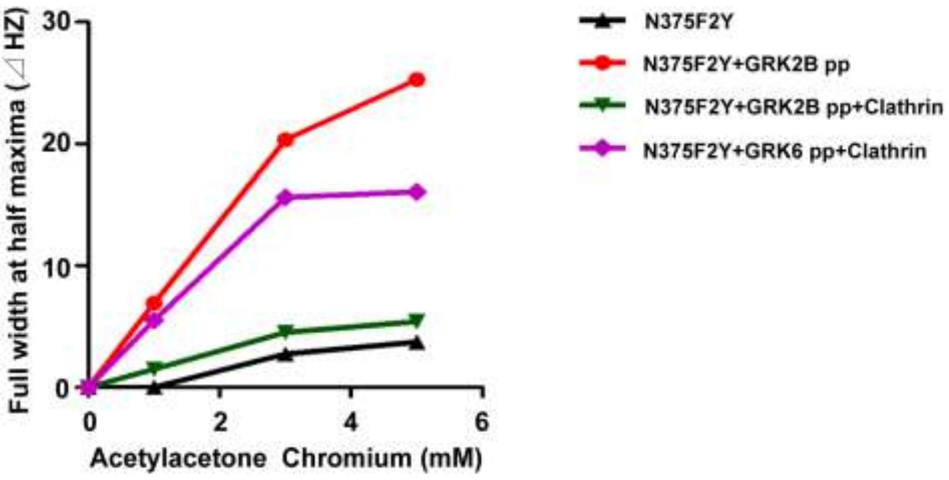

Supplementary Figure 16. Effects of the paramagnetic relaxation agent chromium acetylacetone (Cr) on the n ^{19}F-NMR spectrum of F2Y-N375 of β-arrestin-1 in the presence of different GRK-phospho-peptides or clathrin.

The x-axis represents the concentration of Cr, and the y-axis depicts the increased full width at half maxima (ΔHZ) after the addition of Cr at different concentrations. The increased amplitude of the full width at half maxima in the N375-F2Y-β-arrestin-1/GRK2Bpp complex (red) or (N375-F2Y-β-arrestin-1/GRK6pp complex + clathrin, magnate) is much greater than that of N375-F2Y-β-arrestin-1 alone (black) or the N375-F2Y-β-arrestin-1/GRK2pp/clathrin ternary complex (green) at the N375 position of the ^{19}F-NMR spectrum. These results suggested that the N375 position is more protected from the solvent in β-arrestin-1 alone or the β-arrestin-1/GRK2pp/clathrin ternary complex than in the β-arrestin-1/GRK2Bpp complex.

Supplementary Figure 17.

a

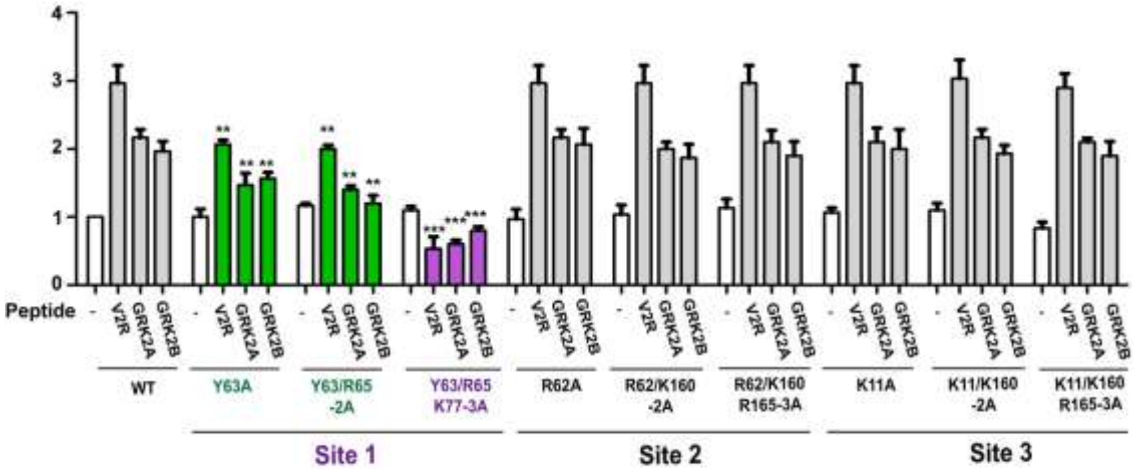

b

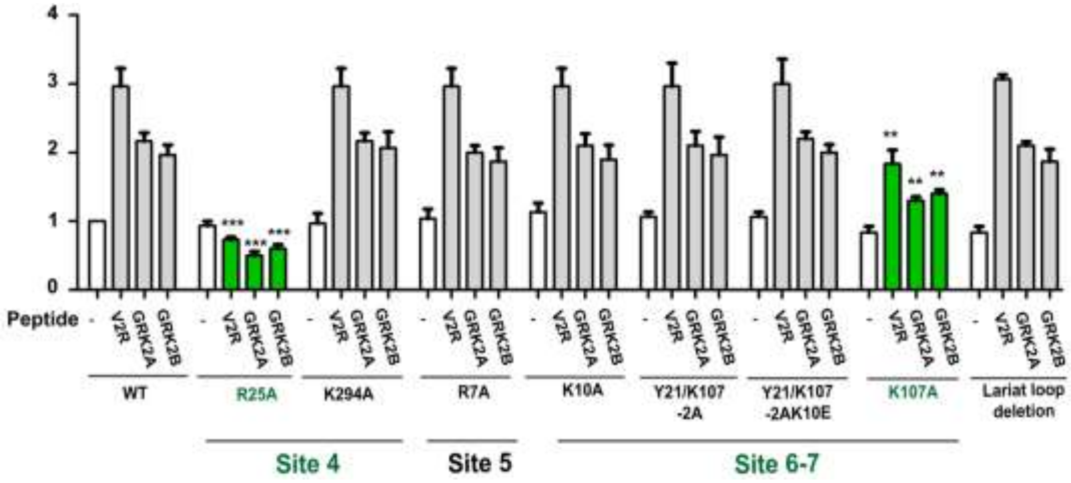

c

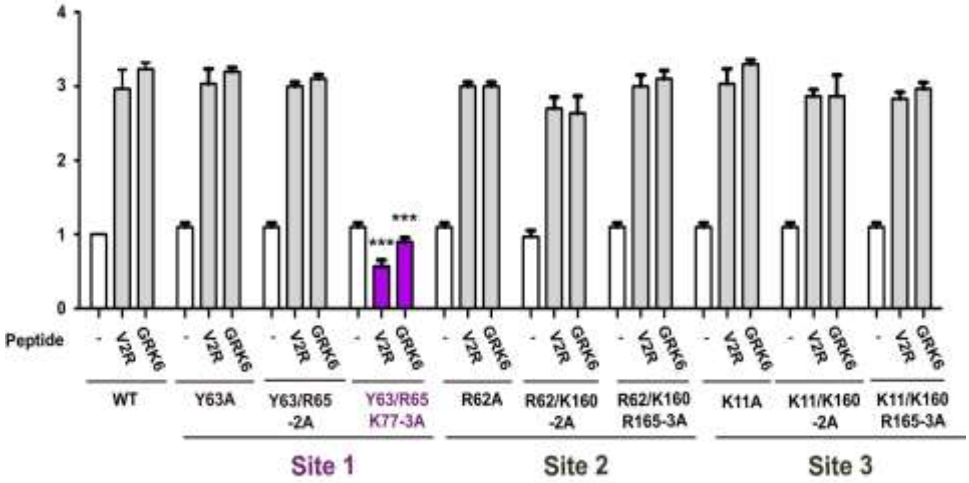

d

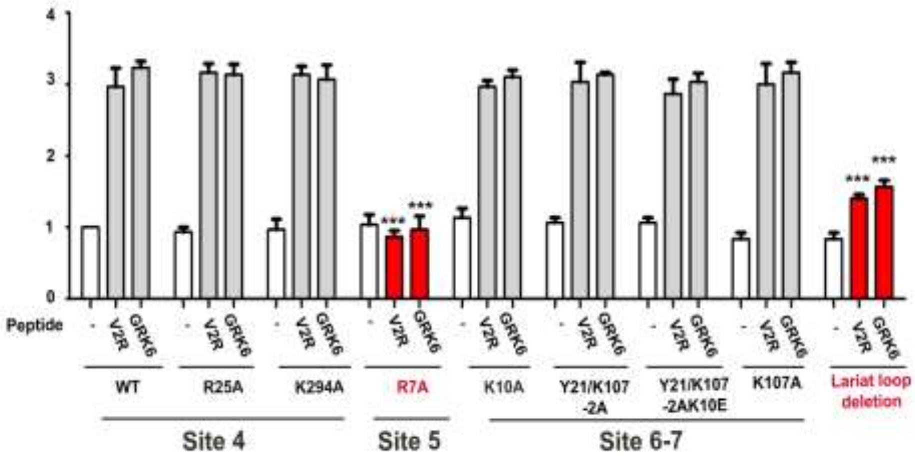

Supplementary Figure 17. Mutation effects on phospho-peptide-induced clathrin or SRC recruitment.

The western blot signals presented in Figure 6A from at least 3 independent experiments were quantified using densitometry and are shown in columns. Green indicates the mutations in β-arrestin-1 that affect phospho-peptide-induced clathrin recruitment; red indicates the mutations that impair the phospho-peptide-stimulated SRC interaction; purple indicates the mutations that abolish both phospho-peptide-induced SRC and clathrin binding. (a) Effects of mutations at phosphate binding sites 1, 2 and 3 on phospho-peptide-induced clathrin recruitment.

(b) Effects of mutations at phosphate binding sites 4, 5, 6/7 and lariat loop deletion on phospho-peptide-induced clathrin recruitment.

(c) Effects of mutations at phosphate binding sites 1, 2 and 3 on the phospho-peptide-induced SRC interaction.

(d) Effects of mutations at phosphate binding sites 4, 5, 6/7 and lariat loop deletion on the phospho-peptide-induced SRC interaction.

(a-d) ***, $p<0.01$; phospho-peptide-induced clathrin/SRC binding with β-arrestin-1 mutants was compared with β-arrestin-1-WT.

Supplementary Figure 18.

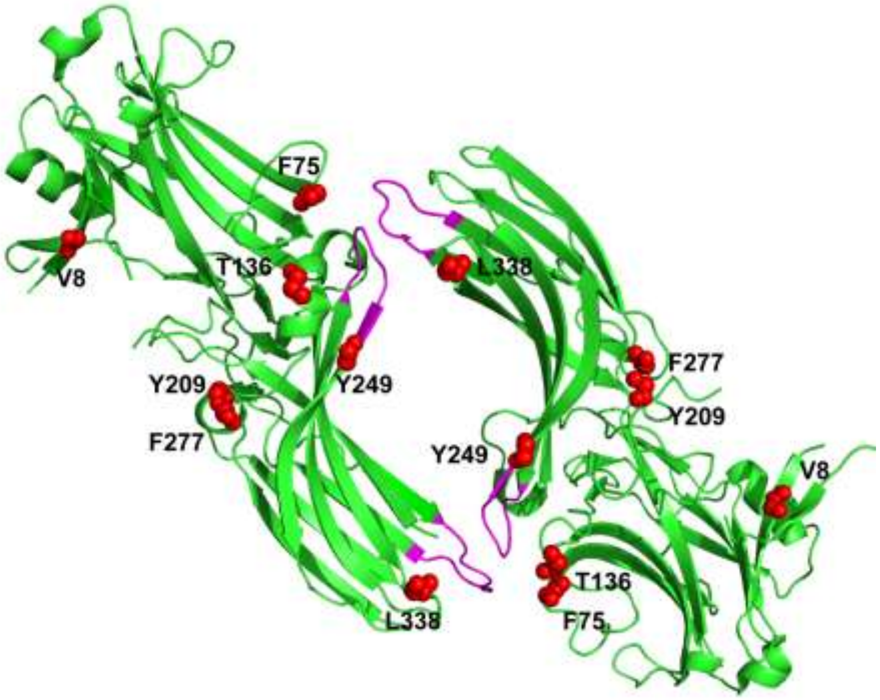

Supplementary Figure 18. Dimer interface of β-arrestin-1 truncation (1-382) (PDB 1G4M). The dimer interface of the truncated β-arrestin-1 (1-382) mainly consists of interactions between two loops, one of which is the loop between R188 and D194 (the loop between β-strands X and XI) in one monomer, and the other is the loop between L243 and Q248 (the loop between β-strands XV and XVI) in another monomer. Among our 17 F2Y-incorporated positions, none are located at the dimer interface, and only Y249 and L338 are close to the dimer interface. It is possible that the changes in the ^{19}F-NMR spectrum at F75- and Y249-incorporated positions could reflect the egulation of propensity of β-arrestin-1 oligomerization by phospho-peptide binding.

Supplementary Figure 19.

a **b**

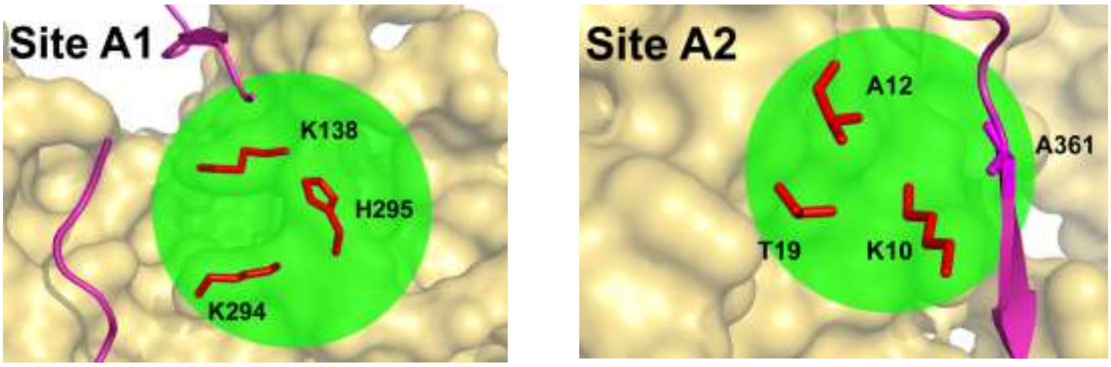

c

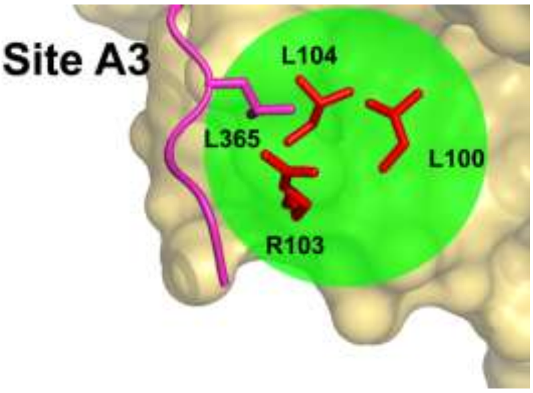

Supplementary Figure 19. Structural representation of other potential phosphate binding sites along the N-terminal phospho-peptide binding concave region of β-arrestin-1, in addition to the 7 phosphate binding sites shown in Figure S2 (PDB: 4JQI).

(a). K138, K294 and H295 constitute a potential phosphate binding site facing an undefined region of V2Rpp in the crystal structure of the V2Rpp/β-arrestin-1 complex.

(b). K10, A12 and T19 constitute a potential phosphate binding site facing the side chain of A361, above phosphate binding site 5.

(c). The L100, R103 and L104 consist a potential phosphate binding site that interact with the L365, also can accommodate a phosphate or a D (Asp).

Supplementary Figure 20.

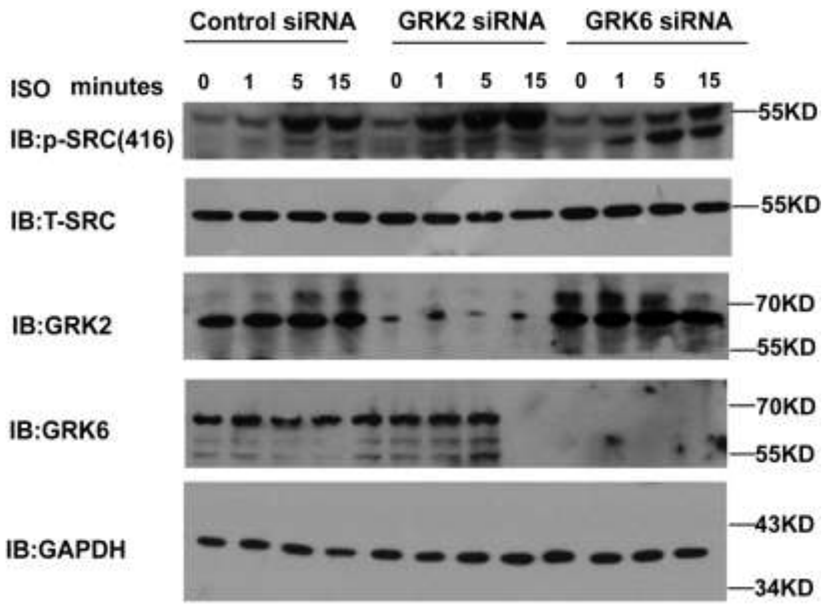

Supplementary Figure 20. Original pictures of western blots in Figure 3b

Supplementary Figure 21.

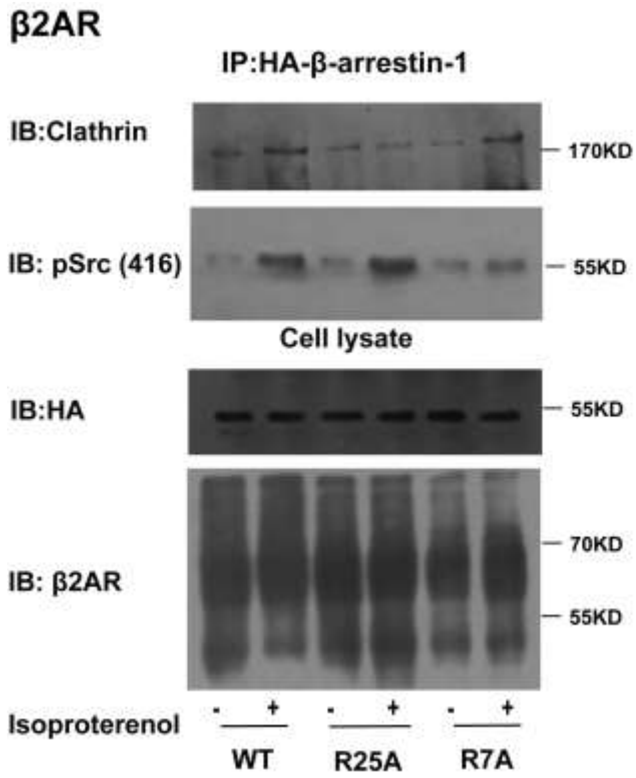

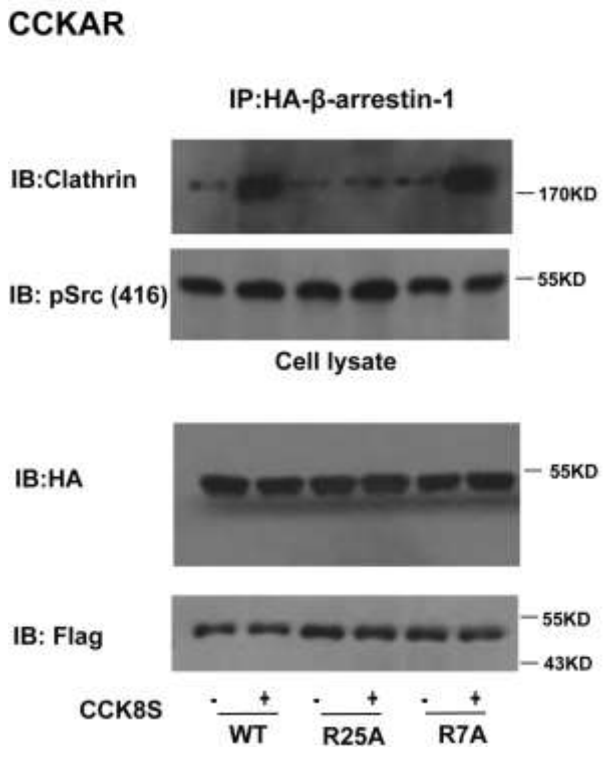

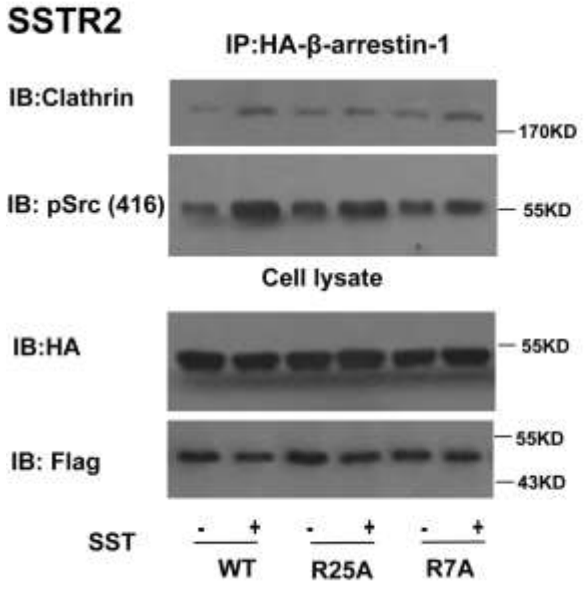

Supplementary Figure 21. Original pictures of western blots in Figure 8a-c

Supplementary Table 1. Chemical shifts in the ^{19}F-NMR spectra assigned to different F2Y incorporation positions in β-arrestin-1.

	Y63F2Y	K138F2Y	K11F2Y	K294F2Y	R7F2Y	Y21F2Y	K107F2Y	V8F2Y
Chemical shift （ppm）	-133.15	-134.43	-134.75	-132.99	-135.15	-130.18	-134.30	-135.26
	F75F2Y	T136F2Y	Y249F2Y	F277F2Y	L338F2Y	N375FY	Y209F2Y	
							Peak I	Peak II
Chemical shift （ppm）	-134.13	-134.38	-134.80	-135.31	-135.99	-135.69	-132.34	-134.29

The NMR spectra of F2Y incorporated β-arrestin-1 at different positions were obtained and the data were processed using 10Hz Lorentzian line broadening and were referenced to the internal TFA standard (-76.5 ppm).

Supplementary Table 2. Effects of the binding of different phospho-peptides on the chemical shifts (Δppm) in the ^{19}F-NMR spectra of the F2Y-phospho-probes of β-arrestin-1

Phospho-peptide	Chemical shift （⊿ppm）							
	Y63F2Y	K138F2Y	R165F2Y	K11F2Y	K294F2Y	R7F2Y	Y21F2Y	K107F2Y
V2Rpp	+1.58	+0.61	+0.32	+0.50	+0.79	+0.27	+5.04	+0.86
V2R5p	-0.04							+0.80
GRK2App	+0.90	-0.02	+0.03	+0.34	+0.56	+0.04	+4.87	+0.26
GRK2Bpp	+0.64	-0.03	+0.02	+0.32	+0.31	-0.04	+4.87	+0.34
GRK6pp	+1.08	+0.01	+0.05	-0.01	+0.03	+0.12	-0.18	+0.02
PKApp	+0.01	+0.02	+0.01	+0.02	+0.01	-0.03	+4.88	+0.17

All of the upfield shifts larger than 0.05 ppm are coloured red. Shifts smaller than 0.05 ppm are coloured black and were not regarded as a significant chemical shift change. Each peak was measured at least twice. The chemical shift for TFA was used as an internal standard (-76.5 ppm).

Supplementary Table 3. Areas or chemical shifts of the^{19}F-NMR spectra for the titration curve of V2Rpp at the Y21 or Y63 position.

β-arrestin-1:V2Rpp	Y21F2Y	Y63F2Y
	Relative peak volume At -135.22 ppm (%)	Chemical shift (⊿ppm)
1:0	0	0
1:0.03	3.18	+0.04
1:0.09	7.41	+0.86
1:3	53.3	+1.58
1:9	54.6	+1.61

Supplementary Table 4. Peak position and volume analysis of the^{19}F-NMR spectra at the Y21-F2Y position of β-arrestin-1 in response to different phospho-peptide binding.

Phospho-peptide	Y21F2Y			
	Peak I		Peak II	
	Chemical shift (ppm)	Relative peakvolume (%)	Chemical shift (ppm)	Relative peakvolume (%)
No pepetide	-130.19	100		0
V2Rpp	-130.18	46.70	-135.22	53.30
GRK2App	-130.17	90.48	-135.04	9.52
GRK2Bpp	-130.18	92.20	-135.05	7.80
GRK6pp	-130.01⊿(-0.18)	100		0
PKApp	-130.17	92.53	-135.05	7.47

The downfield shift larger than 0.05 ppm iscoloured blue

Supplementary Table 5. Effects of the binding of different phospho-peptides on the chemical shifts (Δppm) in the ^{19}F-NMR spectra of F2Y-incorporated positions that do not directly interact with V2Rpp.

Phospho-peptide	Chemical shift （⊿ppm）						
	F75F2Y	T136F2Y	Y209F2Y	Y249F2Y	F277F2Y	L338F2Y	N375F2Y
V2Rpp	-0.62	-0.75	-0.47	-0.63	-0.42	-0.65	-0.95
GRK2App	-0.94	-0.67	-0.03	-0.78	-0.03	-1.06	-0.48
GRK2Bpp	-0.43	-0.51	-0.04	-0.73	-0.01	-0.14	-0.69
GRK6pp	-0.68	-0.68	-0.02	-0.01	-0.26	-0.02	-0.30
PKApp	-0.01	-0.04	-0.02	-0.02	-0.03	-0.03	-0.01

All of the downfield shifts larger than 0.05 ppm are coloured blue. Shifts smaller than 0.05 ppm are coloured black and were not regarded as a significant chemical shift change. Each peak was measured at least twice. The chemical shift of TFA was used as an internal standard (-76.5 ppm).

Supplementary Table 6. Peak position and volume analysis of the^{19}F-NMR spectra at the Y209-F2Y position of β-arrestin-1 in response to different phosphor-peptide binding.

Phospho-peptide	Y209F2Y			
	Peak I		Peak II	
	Chemical shift (ppm)	Relative peakvolume (%)	Chemical shift (ppm)	Relative peakvolume (%)
No pepetide	-132.34	82.75	-134.29	17.25
V2Rpp	-131.87⊿(-0.47)	100		0
GRK2App	-132.31	82.08	-134.26	17.92
GRK2Bpp	-132.30	81.59	-134.25	18.41
GRK6pp	-132.32	82.68	-134.27	17.32
PKApp	-132.32	81.79	-134.27	18.21

The downfield shift larger than 0.05 ppm iscoloured blue

Supplementary Table 7. Effects of the binding of GRK6pp mutants or GRK2Bpp mutants on the chemical shifts (⊿ppm) in the ^{19}F-NMR spectra of F2Y-phospho-probes of beta-arrestin-1.

Phospho-peptide	Chemical shift （⊿ppm）			
	Y63F2Y	K294F2Y	R7F2Y	K107F2Y
GRK6pp	+1.08		+0.12	
GRK2Bpp	+0.64	+0.31		+0.34
GRK6pp-pS355S	+0.02		+0.09	
GRK6pp-E369A	+0.89		+0.01	
GRK2Bpp-pT360T	+0.04	+0.28		
GRK2Bpp-E373A	+0.61	+0.01		
GRK2Bpp-E379/D380A	+0.64			+0.09

All of the upfield shifts larger than 0.05 ppm are coloured red. Shifts smaller than 0.05 ppm are coloured black and were not regarded as a significant chemical shift change. Each peak was measured at least twice. The chemical shift for TFA was used as an internal standard (-76.5 ppm).
